# Extracellular protein analysis of activated sludge and their functions in wastewater treatment plant by shotgun proteomics

**DOI:** 10.1038/srep12041

**Published:** 2015-07-10

**Authors:** Peng Zhang, Yu Shen, Jin-Song Guo, Chun Li, Han Wang, You-Peng Chen, Peng Yan, Ji-Xiang Yang, Fang Fang

**Affiliations:** 1 Key Laboratory of the Three Gorges Reservoir Region’s Eco-Environments of MOE, Chongqing University, Chongqing 400045, China; 2 Key Laboratory of Reservoir Aquatic Environment of CAS, Chongqing Institute of Green and Intelligent Technology, Chinese Academy of Sciences, Chongqing 400714, China

## Abstract

In this work, proteins in extracellular polymeric substances extracted from anaerobic, anoxic and aerobic sludges of wastewater treatment plant (WWTP) were analyzed to probe their origins and functions. Extracellular proteins in WWTP sludges were identified using shotgun proteomics, and 130, 108 and 114 proteins in anaerobic, anoxic and aerobic samples were classified, respectively. Most proteins originated from cell and cell part, and their most major molecular functions were catalytic activity and binding activity. The results exhibited that the main roles of extracellular proteins in activated sludges were multivalence cations and organic molecules binding, as well as in catalysis and degradation. The catalytic activity proteins were more widespread in anaerobic sludge compared with those in anoxic and aerobic sludges. The structure difference between anaerobic and aerobic sludges could be associated with their catalytic activities proteins. The results also put forward a relation between the macro characteristics of activated sludges and micro functions of extracellular proteins in biological wastewater treatment process.

In the surfaces of microbial cell and aggregate, a major player exhibit important physiological functions during the formation and growth of microbial aggregates, which are extracellular polymeric substances (EPS). It is a complex high-molecular-weight mixture of polymers which most main compositions are extracellular proteins and polysaccharides. Exopolysaccharide components have been well elucidated, which mainly includes glucans, fructans alginate, xanthan, Pel and Psl^1^,^2^. Extracellular proteins include enzymes and structural proteins^2^. Biopolymers are broken down to small molecular products by extracellular enzymes in an external digestive system, which are taken up and utilized as carbon and energy sources by microorganisms. Extracellular proteins can promote the attachment and aggregation of suspended flocs into granules and maintain the the stability of the granules^3^,^4^. In addition, extracellular polypeptides and proteins can interact with environmental pollutants, such as restricting the dispersal of nanoparticulate, and adsorbing organic molecules and heavy metal, which serve as an important function in pollutants migration and transformation^5^,^6^,^7^.

Proteins separation and purification from EPS are essential to further investigation because of their complex compositions. Recently, sodium dodecyl sulphate polyacrylamide gel electrophoresis (SDS-PAGE) was used to separate the extracellular proteins, and then the gel pieces were analyzed by mass spectrometry (MS) through further processing^1^,^3^,^8^. However, the humic acids adulterated in protein could interfere or mask the visualization of protein bands in SDS-PAGE^9^. Furthermore, for some special proteins, such as having extremes in molecular mass and isoelectric points and that are hydrophobic or insolvable, the two-dimensional gel electrophoresis/MS method is often difficult to detect. These points render the application of a novel method that is high efficiency and time and labor saving for identifying extracellular protein necessary. Fortunately, shotgun proteomics is an indirect method through measurement amino acid sequences of peptides after proteolytic digestion of intact proteins^10^. By possessing the virtues of more easily fractionated, ionized, and fragmented, shotgun proteomics can be more widely applied for protein analysis^11^. Therefore, this method is much suitable for determining the EPS with low protein concentration and many disruptors. To the best of our knowledge, however, shotgun method has not been used to investigate the extracellular protein in wastewater treatment process.

In this work, the proteins in EPS extracted from the anaerobic, anoxic and aerobic tanks of wastewater treatment plant (WWTP) were separated and purified. The objective of this study is to identify the extracellular proteins by shotgun proteomics, as well as attempt to investigate their origins and functions.

## Results

### Distribution of microbial community

**Figure 1:**
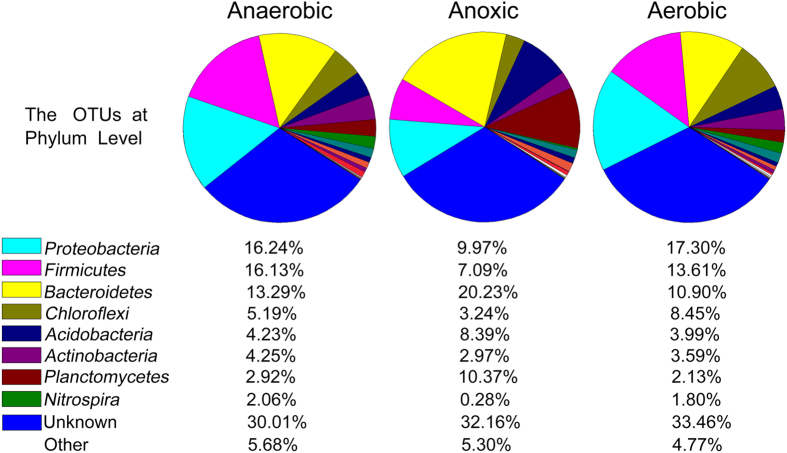
Microbial community structures of anaerobic, anoxic and aerobic sludges at phylum level. Abundance of each phylum was defined as the percentage of the same phylum to the corresponding total sequences for each sample. Some phyla at relative abundance <1% were summarized as other.

In order to search and indentify extracelluar proteins of anaerobic, anoxic and aerobic sludges in database, the operational taxonomic units (OTUs) were obtained at phylum level (Fig. 1). A total of 24 phyla were identified and 22, 20 and 23 for the anaerobic, anoxic and aerobic sludges, respectively. The results show that the *Proteobacteria*, *Firmicutes*, *Bacteroidetes*, *Chloroflexi*, *Acidobacteria*, *Actinobacteria*, *Planctomycetes* and *Nitrospira* were the majority in the three samples. Meanwhile, the classified OTUs and relative abundance were analyzed at genus level to understand the microbial community structure in anaerobic, anoxic and aerobic sludges (Table S1). The dominant functional genuses were *Terrimonas* (8.38%), *Caldilinea* (7.71%), *Nitrospira* (6.89%), *Sulfobacillus* (5.62%), and *Clostridium* (5.53%) in the aerobic sludges sample. *Terrimonas* (23.02%), *Caldilinea* (12.42%), *Pirellula* (7.19%) and *Planctomyces* (6.69%) were the dominant functional genuses in the anoxic sludges sample, whereas *Terrimonas* (11.26%), *Nitrospira* (10.78%), *Clostridium* (10.15%) and *Caldilinea* (5.98%) were the majority in the anaerobic sludges sample. As shown in Table S1, both *Terrimonas* and *Caldilinea* were the dominant genuses in the three samples. Genus *Caldilinea* was the predominantly members of the phylum *Chloroflexi*. *Chloroflexi* play an important structural role in sludge floc formation and can also cause serious sludge bulking or foaming when they are over proliferation^12^. Genus *Nitrosomonas* and *Nitrospira* involved in nitrosation and nitrification processes in biogeochemical nitrogen cycling. However, Genus *Nitrosomonas* in aerobic sludge (0.09%) was much lower than anaerobic (1.12%) and anoxic (1.17%) sludges, which implied that the concentration of ammonia nitrogen in aerobic tank was decreased significantly. Abundant Genus *Nitrospira* revealed that the dominant ammoxidation process was nitrification rather than nitrosation in aerobic tank. Genus *Pirellula* and Genus *Planctomyces* belong to phylum *Planctomycetes*, and they are obligate aerobic bacteria. Many aerobic bacteria were present in anaerobic and anoxic tank, which could be associated with the influent substrate and a few aerobic areas existed in these tanks.

### FT-IR spectra characterization

The FT-IR spectra of extracelluar proteins from anaerobic, anoxic and aerobic sludges are shown in Figure S1. The IR bands were assigned according to the existing literatures^13^,^14^,^15^. The bands at 3431 and 3268 cm^−1^ were assigned to the O–H or N–H stretching vibrations. The peak at 1654 cm^−1^ was assigned to the C = O stretching vibration of amide I. The N–H deformation vibration of amide II was observed at 1532 cm^−1^, whereas the peak at 1230 cm^−1^ was due to the C–N stretching vibration of amide III. The bands at 1131 cm^−1^, 1082 cm^−1^, 1041 cm^−1^, and 983 cm^−1^ were attributed to the C–O and C–C stretching as well as the C–O–C and C–O–H deformation vibrations of the polysaccharide structures. The bands at 1082 cm^−1^ and 1041 cm^−1^ may be also associated with the P = O stretching, C–P–O and C–O–H stretching vibrations, respectively. The peak at 983 cm^−1^ was also likely to be related to O–P–O stretches. The results indicated that the extracellular protein samples could mix/bind carbohydrate and phosphorylated macromolecules^14^.

### Distributions of isoelectric points (pI) and molecular mass

The MS/MS spectra of protein samples were automatically searched against the protein database according to the dominant phyla of microbial communities and 149, 129 and 131 proteins were identified in anaerobic, anoxic and aerobic sludge samples, respectively. Figure S2 shows the distributions of theoretical molecular mass and pI of the indentified proteins in the anaerobic, anoxic and aerobic sludge samples. The theoretical molecular mass of most proteins were ranged from 10 kDa to 100 kDa. In fact, a large number of components with molecular mass less than 10 kDa present in EPS, which attributed to the protein hydrolysis and non-protein presented in EPS^16^. pI of the three protein samples were ranged from 4 to 12. Most pI ranged from 5 to 6, which indicated that the major proteins in EPS were negative charge. It was consistent with the result of zeta potential of EPS^17^.

### Origin and function of protein

**Figure 2:**
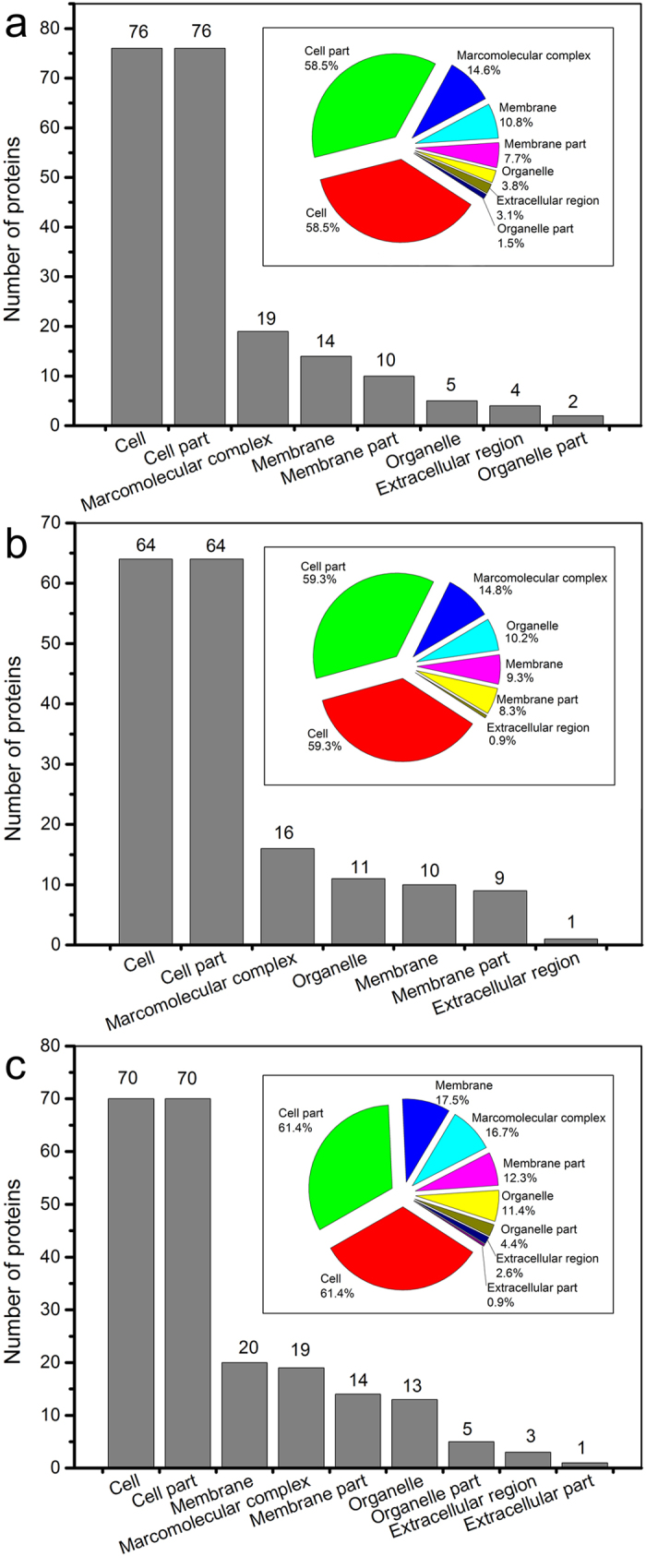
Proteins classification according to cellular component. Numbers and percentages (inset) of the identified proteins in anaerobic (**a**), anoxic (**b**) and aero**b**ic (**c**) sludge samples.

130, 108 and 114 proteins in the identified proteins of anaerobic, anoxic and aerobic sludge samples were classified, respectively, according to GOA in terms of biological process, cellular component and molecular function^18^. The classified protein names and microorganism assignments are listed in Table S2-S4. The qualitative FT-IR spectra of three extracellular protein samples are similar, but only 11 shared proteins were present in the three samples. Indeed, the amounts of the shared proteins were 17, 23 and 23 in anaerobic and anoxic, anaerobic and aerobic, and anoxic and aerobic, respectively (Table S5). These differences could be attributed to the various microorganisms and influents in 3 tanks. Figure S3 shows the proteins in the three samples involved 13 biological processes related to metabolic process, celluar process and single-organis process, etc. As shown in Fig. 2, the identified proteins were localized in cell, cell part, marcomolecular complex, membrane, membrane part, organelle, organelle part, extracellular region and extracellular part. The results showed that the identified proteins widely distributed, and a kind of protein may exist in multiple parts of the cell. The anaerobic, anoxic and aerobic sludge samples obtained 8, 7 and 9 catalogues of cellular component, respectively. Most proteins (58.5%, 59.3% and 61.4% for anaerobic, anoxic and aerobic sludge samples, respectively) primarily located in the cell and cell part.

**Figure 3:**
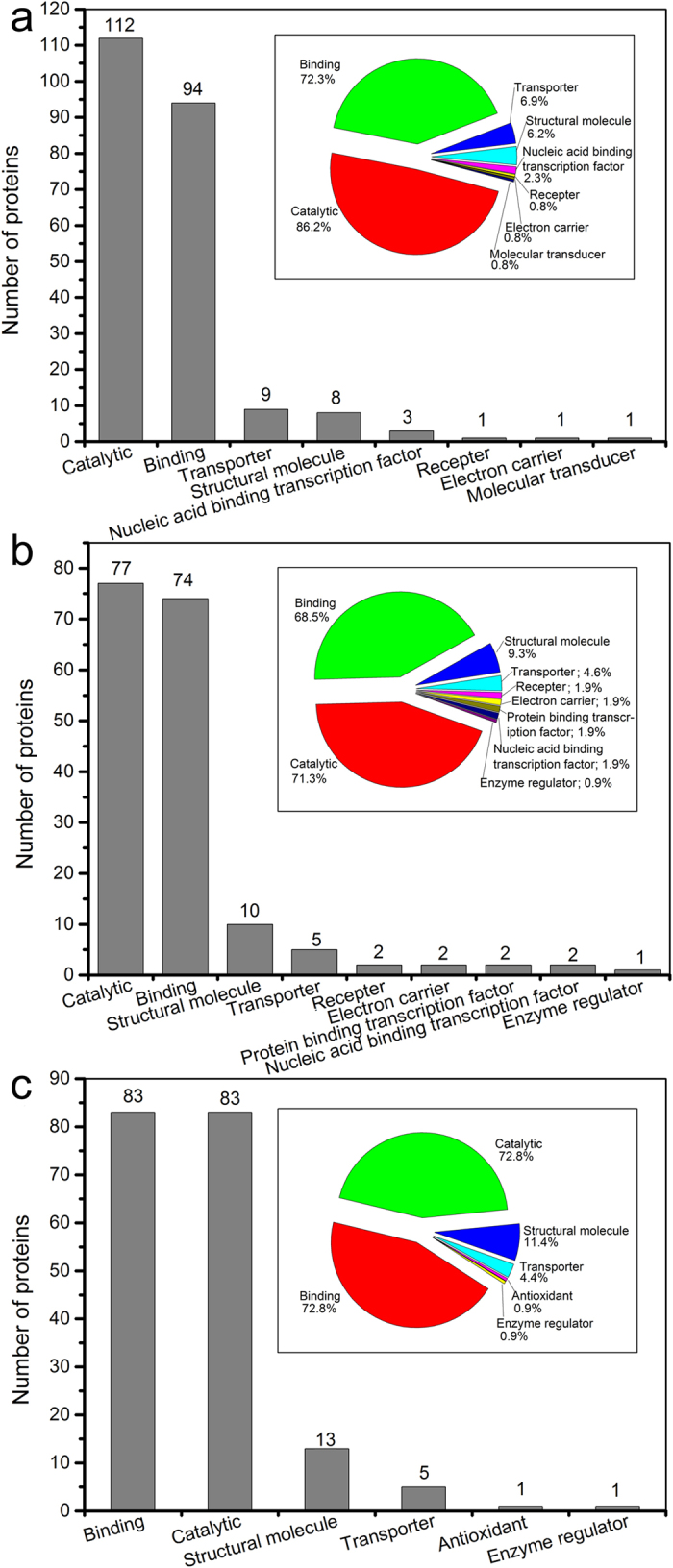
Proteins classification according to molecular function. Numbers and percentages (inset) of the identified proteins in anaerobic (**a**), anoxic (**b**) and aero**b**ic (**c**) sludge samples.

**Figure 4:**
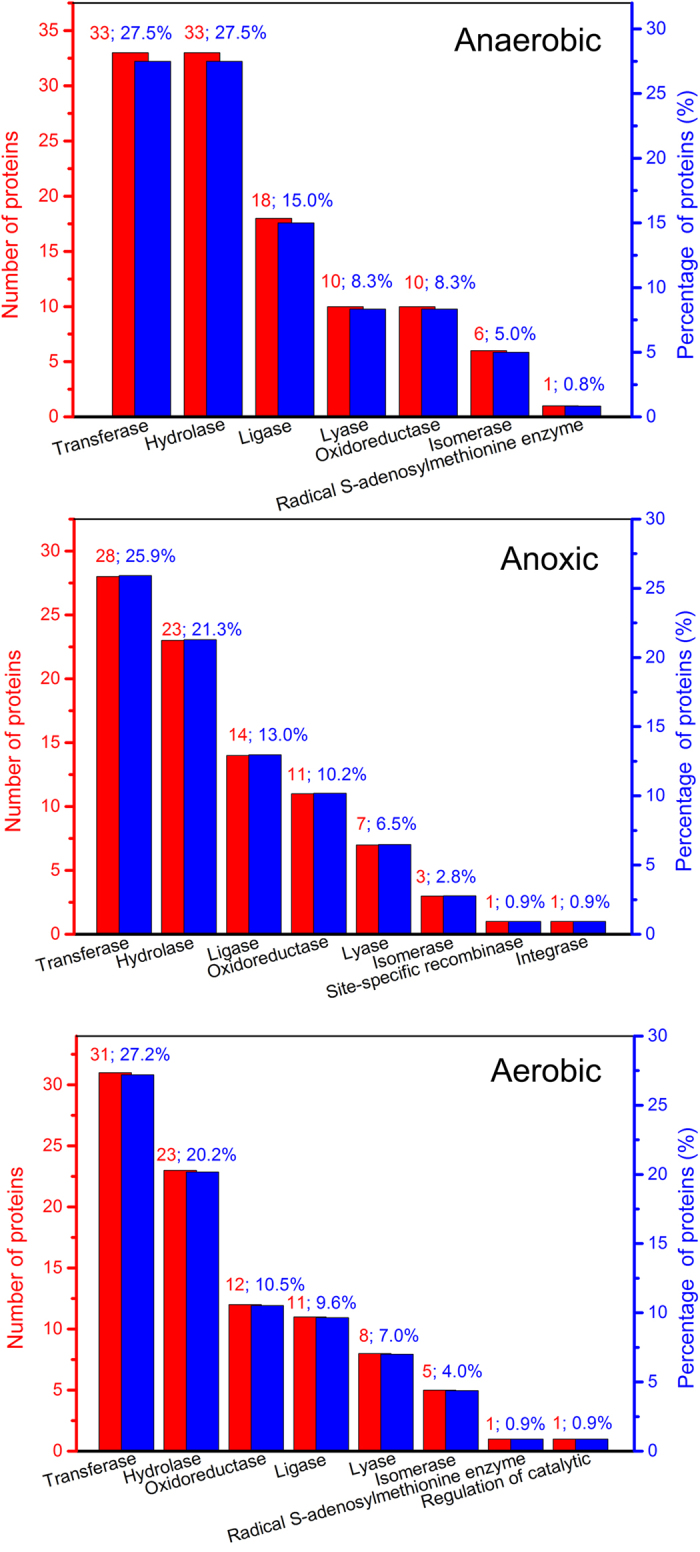
Numbers and percentages of the classified proteins related to catalytic activity in anaerobic, anoxic and aerobic sludge samples.

**Figure 5:**
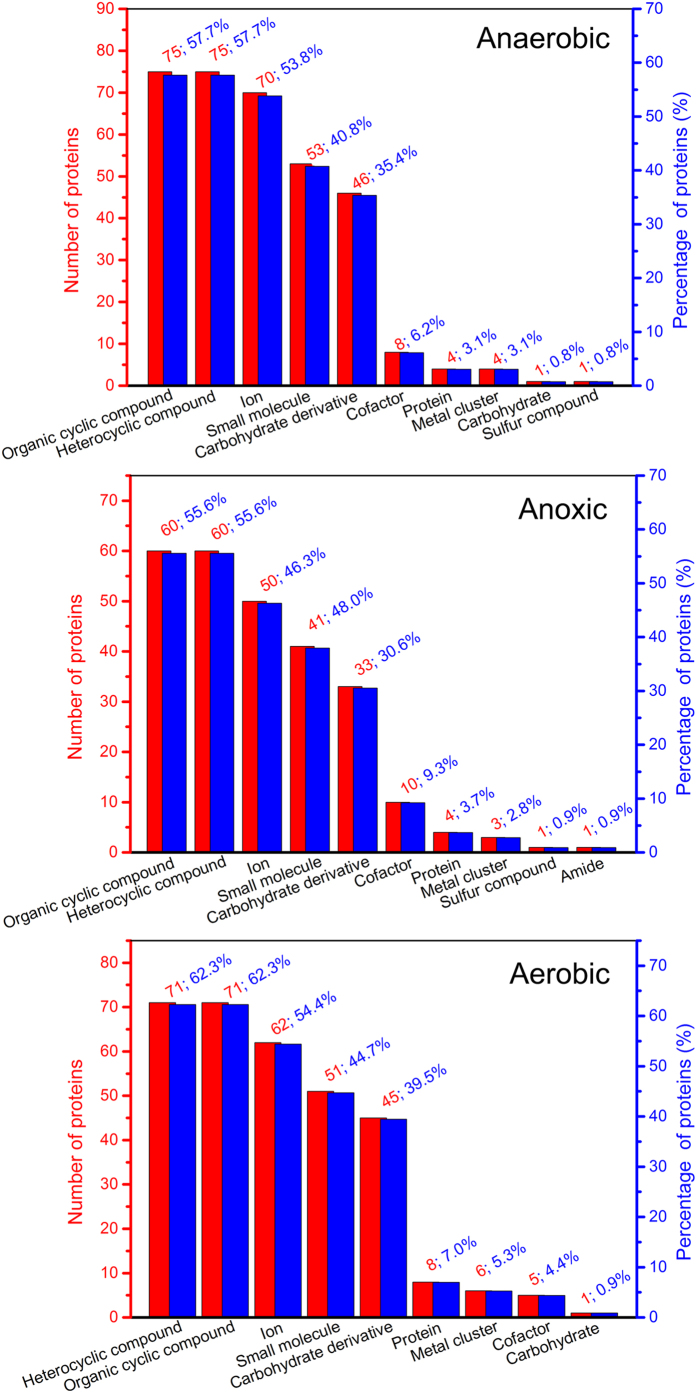
Numbers and percentages of the classified proteins related to binding activity in anaerobic, anoxic and aerobic sludge samples.

Figure 3 shows a broad range of molecular functions of proteins, including catalytic activity, binding activity, structural molecule activity, transporter activity, and so on. 8, 9 and 6 catalogues of molecular function of anaerobic, anoxic and aerobic sludge samples were classified, respectively, and a kind of protein could also have multiple molecular functions. The most major molecular functions were catalytic activity (86.2%, 71.3% and 72.8% for anaerobic, anoxic and aerobic sludge samples, respectively) and binding activity (72.3%, 68.5% and 72.8% for anaerobic, anoxic and aerobic sludge samples, respectively). The proteins from anaerobic, anoxic and aerobic sludge samples in the catalytic activity group were further classified into 7, 8 and 8 subgroups, respectively (Fig. 4). Most proteins were relevant to transferase activity and hydrolase activity. The proteins related to binding acitivity were also further classified into 10, 10 and 9 subgroups for anaerobic, anoxic and aerobic sludge samples, respectively, and organic cyclic compound binding, heterocyclic compound binding, ion binding, small molecule binding, carbohydrate derivative binding were the most majority (Fig. 5).

4 (3.1%), 1 (0.9%) and 3 (2.6%) proteins in anaerobic, anoxic and aerobic sludge samples were derived from extracellular region, respectively, and the identified names are exhibited in Table 1. tRNA N6-adenosine threonylcarbamoyltransferase involves in proteolysis and tRNA processing, which is related to transferase activity (transferring acyl groups), metalloendopeptidase activity and iron ion binding activity. Enolase is associated with phosphopyruvate hydratase activity and magnesium ion binding activity, and participates in glycolytic process. Bifunctional hemolysin/adenylate cyclase has calcium- and calmodulin-responsive adenylate cyclase activity, as well as nucleotide, ATP, calmodulin and calcium ion binding activity, which involved in cell killing process. Adenosine monophosphate-protein transferase and cysteine protease IbpA involves in the molecular functions of cysteine-type endopeptidase, nucleotidyltransferase and protein adenylyltransferase, as well as nucleotide and ATP binding. It implied that these extracellular proteins played potential roles in binding multivalence cations and organic moleculars, as well as in macromolecules hydrolysis. For the proteins of anoxic sludge sample derived from extracellular region, which molecular function is unknow. D-(-)-3-hydroxybutyrate oligomer hydrolase and IgA-specific serine endopeptidase autotransporter exhibit hydroxybutyrate-dimer hydrolase activity and serine-type endopeptidase activity, respectively. The results demonstrated that the anaerobic and aerobic sludges had various extracellular enzymes and played different roles.

## Discussion

Most extracellular proteins primarily located in the cell and cell part, which implied that the main extracelluar proteins were derived from cell lysis. It was consistent with the result described by Silva *et al*.^9^. Some enzymes in EPS could remain catalytic activity under appropriate conditions and play relevant roles in extracellular environment^19^,^20^,^21^. Figure 4 illustrates that the proteins related to catalytic activity from anaerobic (112, 86.2%) sludge sample were much more than those from anoxic (77, 71.3%) and aerobic (83, 72.8%) sludge samples. Similarly, the results also showed that the proteins associated with hydrolase activity from anaerobic sludge sample (33) were also much more abundant, compared wtih those from anoxic and aerobic sludge samples (23 and 23, respectively). More proteins related to catalytic activity and hydrolase activity in anaerobic sludge could promote the catalysis and degradation of nutrition matrix^22^. The results demonstrated that many extracelluar proteins had the binding sites of metal ions, and inorganic or organic molecules. The FT-IR spectra of extracellular proteins also revealed that some carbohydrates and nucleic acids could bind with proteins. Many previous studies showed the high binding activity of EPS toward metal ions, dyes and pharmaceutical^23^,^24^,^25^,^26^. Figure 5 shows the protein amounts related to binding activity (organic molecules and ions) in anaerobic sludge and aerobic sludge were similar, whereas they were more abundant than those in anoxic sludge. It indicated that anaerobic sludge proteins and aerobic sludge proteins could have more excellent pollutants removal than anoxic sludge proteins by biosorption.

**Figure 6:**
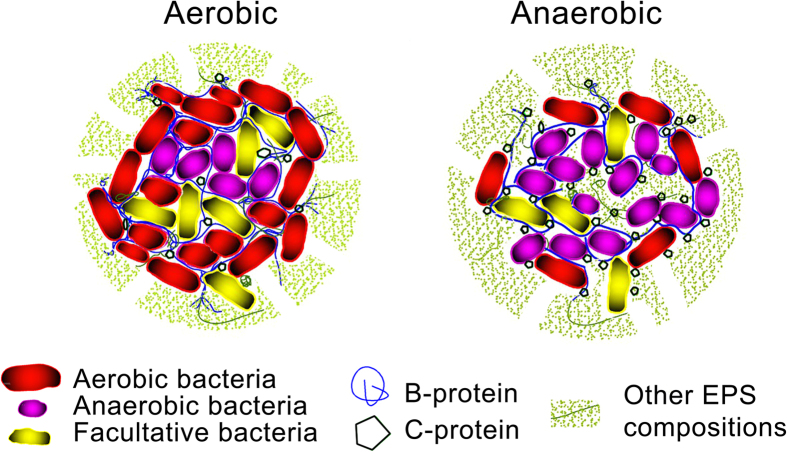
A proposed schematic of the floc structures of anaerobic and aerobic sludges (B-protein, binding activity proteins; C-protein, catalytic activity proteins).

Anaerobic and aerobic sludges exhibit distinct aggregate architectures, biochemical processes, and surface characteristics. Anaerobic condition creates porous activated sludge, whereas aerobic condition produced more compact flocs^27^. Influent substrates, microorganisms and biochemical processes were different in the two sludges. Aerobic sludge displays higher aggregation rate and flocculation potential than anaerobic sludge^28^,^29^. Genus *Clostridium* mainly contains a large group of anaerobic bacillus, which was also abundant in aerobic sludge. The presence of the anaerobic environment could be ascribed to the tightly aggregation of microorganisms. It indicated that the two sludges’ structures were various, and a probable reason was the diversities of catalytic activity proteins in the sludges. Excessive extracellular enzymes could deteriorate the stability of aggregate structures from the following probably aspects: (1) EPS was hydrolyzed or degraded, causing to EPS fracture and aggregate structures damage, which resulted to sludge floc dispersion; (2) Microbial attachment was suppressed, reducing the sludge floc compactness^4^,^30^,^31^,^32^. Both the amount and proportion of catalytic activity proteins in anaerobic sludge were higher than those in aerobic sludge. Furthermore, the structural molecule proteins in aerobic sludge were more abundant compared with that in anaerobic sludge. Based on these results, Fig. 6 exhibits a hypothetical schematic of the floc structures of anaerobic and aerobic sludges from the perspective of the catalytic activity proteins. The aerobic sludge forms densely floc and anaerobic bacteria were wraped in the interior by aerobic bacteria, whereas the anaerobic sludge exhibits loosely floc.

## Conclusions

Extracellular proteins extracted from WWTP sludges were identified by shotgun proteomics, and 130, 108 and 114 proteins were classified in anaerobic, anoxic and aerobic sludges, respectively. Most proteins originated from cell and cell part, and their most major molecular functions were catalytic activity and binding activity, indicating that their main roles in activated sludges were multivalence cations and organic matters binding, as well as catalysis and degradation. Both the amount and proportion of catalytic activity proteins in anaerobic sludge were higher than those in aerobic sludge. The results implied that the catalytic activity proteins in anaerobic sludge and aerobic sludge contributed to their different floc structures.

## Methods

### Sludge and Illumina MiSeq sequencing

Activated sludges were collected from the anaerobic, anoxic and aerobic tanks of Jiguanshi WWTP, Chongqing, China. The samples were stored in darkroom and at 4 °C before DNA and EPS extraction. DNA was extracted using 3S column centrifugal environmental sample DNA extraction kit (Shennengbocai Biotech, Shanghai, China). 16S rRNA gene polymerase chain reaction amplification, Illumina MiSeq sequencing, and data analysis were performed by Personalbio Biotech (shanghai, China) Co., Ltd. Primers 520F (5-AYTGGGYDTAAAGNG-3) and 802R (5-TACNVGGGTATCTAATCC-3) which target V4 regions of bacterial 16S rRNA genes were selected.

### EPS extraction and protein purification

The EPS in activated sludge was extracted using cationic exchange resin (CER) method. In general, 200 mL of sludge suspention and CER (70 g/g VSS) were transferred to a conical flask. The extraction was carried out in a shaking incubator at 250 rpm for 2 h at 4 °C. Then the suspension was centrifuged at 10000 *g* for 20 min at 4 °C. At last, the supernatant was filtered through 0.45 μm filter and stored at 4 °C for further experiments.

The protein in EPS was separated by the modified method of trichloroacetic acid (TCA) precipitation^9^. The TCA (100%, w/v) was added to the EPS sample solution to a final concentration of 13%, and the mixture was incubated overnight at 4 °C after a well mixed. Then the mixture was centrifuged at 13000 *g* for 20 min at 4 °C, and the supernatant was removed. The residual TCA in the precipitates was washed using acetone and centrifuged at 12000 *g* for 3 min, and the supernatant was rejected. The washing step was performed 3 times at 4 °C. At last, the precipitates were collected for enzymatic hydrolysis.

### Protein digestion

Extracellular protein digestion was conducted according to the method of Wisniewski *et al*.^33^. In general, approximately 30 μg of the protein pellet was dissovled in 30 μl of SDT buffer (4% SDS, 100 mM dithiothreitol and 150 mM Tris-HCl, pH 8.0) at 90 °C for 5 min. The detergent, dithiothreitol and other small molecules were discarded using 200 μl of UA buffer (8 M urea in 150 mM Tris-HCl, pH 8.0) by repeated ultrafiltration (30 kD). Then 100 μl of 0.05 M iodoacetamide in UA buffer was added to block reduced cysteine residues, and the samples were incubated for 20 min in darkness at room temperature. The filter was washed with 100 μl of UA buffer three times and followed by 100 μl of 25 mM NH_4_HCO_3_ twice. Finally, the protein suspension with 2 μg of trypsin in 40 μl of 25 mM NH_4_HCO_3_ incubated overnight at 37 °C to protein digestion. The resulting peptides were filtrated and collected for further analysis.

### Liquid chromatography (LC)-MS/MS

The LC system (Ettan MDLC, GE Healthcare, USA) was used for desalting and separation of tryptic peptides mixtures. In this system, samples were desalted on reverse phase (RP) trap columns (Zorbax 300 SB C18, Agilent Technologies, USA), and then separated on a RP column (150 μm × 100 mm, Fremont Column Technology Inc., USA). The mobile phase A was 0.1% formic acid in HPLC-grade water and the mobile phase B was 0.1% formic acid in acetonitrile. 20 μg of tryptic peptide mixtures was separated at a flow rate of 2 μL/min with a linear gradient (4–50% solution B, 50 min; 50–100% solution B, 4 min; 100% solution B, 6 min) in the columns. A mass spectrometer (LTQ Velos, Thermo Scientific, USA) was equipped with a micro-spray interface and connected to the LC setup to detecte the eluted peptides. The MS/MS spectra were set so that one full scan mass spectrum (*m/z* 300–1800) was followed by twenty MS/MS events of the most intense ions using dynamic exclusion (repeat count 2, repeat duration 30 seconds, exclusion duration 90 seconds).

### Data analysis

MS/MS spectra were automatically searched against the protein database based on the dominant phyla of microbial communities (*Verrucomicrobia*, *Proteobacteria*, *Planctomycetes*, *Nitrospira*, *Gemmatimonadetes, Firmicutes*, *Deinococcus-Thermus*, *Chloroflexi*, *Bacteroidetes*, *Actinobacteria*, *Acidobacteria*) using the BioworksBrowser rev. 3.1 (Thermo Electron, San Jose, USA). Protein identification results were extracted from SEQUEST out files with BuildSummary. The mass tolerance allowed for the precursor ions was 2.0 Da and fragment ions was 0.8 Da, respectively. The protein identification criteria were based on Delta CN (≥0.1) and cross-correlation scores with the minimum values of Xcorr: one charge ≥1.9, two charges ≥2.2, and three charges ≥3.75^34^. Some low abundance proteins in the samples and the insufficiency of protein database could restrict the protein identification. In order to obtain more abundant proteins in our samples, in this work, the protein were indentified at least 1peptide^35^,^36^,^37^. Classifications of identified proteins were conducted using gene ontology annotation (GOA; http://www.uniprot.org/uniprot/) according to the protein accession numbers.

### Fourier transform infrared spectra (FT-IR)

The FT-IR of extracellular protein was obtained by an FT-IR spectrophotometer (Tensor 27, Bruker, Germany). The extracellular protein precipitates were lyophilized and mixed with IR grade KBr powders to prepare the pellets to measure.

## Additional Information

**How to cite this article**: Zhang, P. *et al*. Extracellular protein analysis of activated sludge and their functions in wastewater treatment plant by shotgun proteomics. *Sci. Rep*. **5**, 12041; doi: 10.1038/srep12041 (2015).

## Supplementary Material

Supplementary Information

## Figures and Tables

**Table 1:** Proteins localized in extracellular region.

Sludge samples	Protein description
Anaerobic	tRNA N6-adenosine threonylcarbamoyltransferase
	Enolase
	Bifunctional hemolysin/adenylate cyclase
	Adenosine monophosphate-protein transferase and cysteine protease IbpA
Anoxic	ESX-1 secretion-associated protein EspB
Aerobic	Enolase
	D-(-)-3-hydroxybutyrate oligomer hydrolase
	IgA-specific serine endopeptidase autotransporter
