# Supplementary material for: Extracellular protein analysis of activated sludge and their functions in wastewater treatment plant by shotgun proteomics
Source: Sci Rep. 2015 Jul 10;5:12041. doi: 10.1038/srep12041 (PMC4498230; doi:10.1038/srep12041)
Supplement: Supplementary Information [file srep12041-s1.pdf]

Supplementary information

Extracellular protein analysis of activated sludge and their functions in wastewater treatment plant by shotgun proteomics

Peng Zhang[1], Yu Shen[2], Jin-Song Guo[1,2], Chun Li[1], Han Wang[1] You-Peng Chen[1,2,*], Peng Yan[2], Ji-Xiang Yang[2], Fang Fang[1,*]

[1] Key Laboratory of the Three Gorges Reservoir Region's Eco-Environments of MOE, Chongqing University, Chongqing 400045, China

[2] Key Laboratory of Reservoir Aquatic Environment of CAS, Chongqing Institute of Green and Intelligent Technology, Chinese Academy of Sciences, Chongqing 400714, China

*Corresponding Author

Prof. You-Peng Chen

Fax: +86-23-65935818, Tel: +86-23-65935818

Email: ypchen@cigit.ac.cn

Prof. Fang Fang

Fax: +86-23-65127370, Tel: +86-23-65127370

Email: fangfangcq@cqu.edu.cn

Table S1. Classified OTUs in anaerobic, anoxic and aerobic sludges at genus level (relative abundance > 1%)

Table S2. The classified extracellular proteins in anaerobic sludge analyzed by shotgun method.

Table S3. The classified extracellular proteins in anoxic sludge analyzed by shotgun method.

Table S4. The classified extracellular proteins in aerobic sludge analyzed by shotgun method.

Table S5. The shared proteins in three sludge samples.

Figure S1. FT-IR spectra of extracelluar proteins from anaerobic, anoxic and aerobic sludges

Figure S2. Distributions of pI and molecular mass of the extracelluar proteins in anaerobic, anoxic and aerobic sludges.

Figure S3. Proteins classification according to biological process. Numbers and percentages of the identified proteins in anaerobic, anoxic and aerobic sludge samples.

Table S1. Classified OTUs in anaerobic, anoxic and aerobic sludges at genus level (relative abundance > 1%)

Phylum	Genus	Aerobic	Anoxic	Anaerobic
Bacteroidetes	*Terrimonas*	8.38	23.02	11.26
Chloroflexi	*Caldilinea*	7.71	12.42	5.98
Nitrospira	*Nitrospira*	6.89	1.57	10.78
Firmicutes	*Sulfobacillus*	5.62	0.00	0.00
Firmicutes	*Clostridium*	5.53	4.31	10.15
Proteobacteria	*Novosphingobium*	4.28	3.77	2.79
Firmicutes	*Cerasibacillus*	4.13	0.00	0.00
Actinobacteria	*Bifidobacterium*	3.46	2.49	3.52
Spirochaetes	*Turneriella*	2.92	0.64	3.54
Proteobacteria	*Thauera*	2.89	0.11	2.05
Firmicutes	*Streptococcus*	2.35	0.18	0.92
Firmicutes	*Ureibacillus*	2.29	0.00	0.00
Chloroflexi	*Longilinea*	2.28	0.07	1.66
Nitrospira	*Leptospirillum*	2.20	0.00	0.00
Euryarchaeota	*Methanospirillum*	2.19	0.96	2.02
Proteobacteria	*Desulfocapsa*	1.68	0.85	1.62
Firmicutes	*Lactococcus*	1.39	0.00	0.08
Proteobacteria	*Acidisoma*	1.34	0.46	2.06
Proteobacteria	*Acinetobacter*	1.33	0.78	1.20
Firmicutes	*Guggenheimella*	1.33	0.07	2.02
Proteobacteria	*Desulfobulbus*	1.30	1.28	1.63
Proteobacteria	*Zoogloea*	1.27	0.00	0.46
Planctomycetes	*Planctomyces*	1.20	6.69	1.76
Proteobacteria	*Geobacter*	1.15	0.18	1.23
Planctomycetes	*Pirellula*	1.09	7.19	1.75
Verrucomicrobia	*Prosthecobacter*	0.98	1.60	1.33
Actinobacteria	*Nocardioides*	0.89	1.00	0.71
Proteobacteria	*Hyphomicrobium*	0.88	1.71	1.29
Euryarchaeota	*Methanosaeta*	0.86	3.74	1.78
Proteobacteria	*Arcobacter*	0.72	0.21	1.35
Proteobacteria	*Bradyrhizobium*	0.72	2.74	1.84
Firmicutes	*Blautia*	0.70	1.21	1.43
Firmicutes	*Turicibacter*	0.61	0.25	1.23
Actinobacteria	*Conexibacter*	0.57	0.39	1.03
Planctomycetes	*Zavarzinella*	0.48	2.60	0.79
Planctomycetes	*Blastopirellula*	0.38	2.46	0.39
Proteobacteria	*Nitrosomonas*	0.09	1.17	1.12

Table S2. The classified extracellular proteins in anaerobic sludge analyzed by shotgun method.

No.	Protein description	Peptide counts	Unique peptide counts	Cover percent	Molecular mass	PI	Microorganism species
1	Exodeoxyribonuclease 7 small subunit	1	1	27.71%	9259.45	4.73	*Rhizobium meliloti* (strain 1021)
2	10 kDa chaperonin	1	1	20.62%	10426.08	9	*Buchnera aphidicola subsp. Cinara cedri* (strain Cc)
3	30S ribosomal protein S15	1	1	17.58%	10594.29	10.33	*Rickettsia africae* (strain ESF-5)
4	50S ribosomal protein L24	1	1	16.85%	9988.86	10.91	*Chlorobium chlorochromatii* (strain CaD3)
5	Probable Fe^{2+}-trafficking protein	1	1	15.22%	10789.52	7.86	*Anaeromyxobacter dehalogenans* (strain 2CP-C)
6	50S ribosomal protein L28	1	1	14.61%	10072.14	11.57	*Chlamydophila abortus* (strain DSM 27085 / S26/3)
7	ATP synthase subunit b	1	1	13.04%	20614.99	9.18	*Myxococcus xanthus* (strain DK 1622)
8	ATP synthase subunit beta	5	4	12.23%	50654.16	5.31	*Aromatoleum aromaticum* (strain EbN1)
9	3-dehydroquinate dehydratase	1	1	11.64%	15762.85	4.94	*Corynebacterium diphtheriae* (strain ATCC 700971 / NCTC 13129 / Biotype gravis)
10	50S ribosomal protein L17	1	1	10.71%	15551.08	9.92	*Magnetospirillum magneticum* (strain AMB-1 / ATCC 700264)
11	50S ribosomal protein L14	1	1	10.66%	13174.28	9.91	*Oenococcus oeni* (strain ATCC BAA-331 / PSU-1)
12	Regulatory protein RecX	1	1	10.56%	17864.04	5.73	*Halorhodospira halophila* (strain DSM 244 / SL1)
13	Peptide deformylase	1	1	10.40%	19361.31	4.77	*Caulobacter* sp. (strain K31)
14	Putative pterin-4-alpha-carbinolamine dehydratase	1	1	10.17%	13321.12	5.93	*Azotobacter vinelandii* (strain DJ / ATCC BAA-1303)
15	Malate dehydrogenase	2	2	10.06%	35000.12	6.24	*Dechloromonas aromatica* (strain RCB)
16	50S ribosomal protein L16	1	1	9.49%	15119.97	11.11	*Desulfovibrio salexigens* (strain ATCC 14822 / DSM 2638 / NCIB 8403 / VKM B-1763)
17	Probable inorganic	1	1	9.00%	32682.63	6.53	*Alcanivorax borkumensis* (strain SK2 / ATCC 700651 / DSM

	polyphosphate/ATP-NAD kinase						11573)
18	Thiamine-phosphate synthase	1	1	8.92%	22699.61	4.63	*Erythrobacter litoralis* (strain HTCC2594)
19	Diaminopimelate epimerase	1	1	8.83%	31230.65	6.14	*Bartonella tribocorum* (strain CIP 105476 / IBS 506)
20	Putative Holliday junction resolvase	1	1	8.70%	15025.32	5.47	*Bacillus pumilus* (strain SAFR-032)
21	Glycerol-3-phosphate acyltransferase	1	1	8.59%	20686.75	10.33	*Bradyrhizobium* sp. (strain BTAi1 / ATCC BAA-1182)
22	Urease accessory protein UreF	1	1	8.53%	21979	5.77	*Mycobacterium marinum* (strain ATCC BAA-535 / M)
23	50S ribosomal protein L25	1	1	8.48%	24460.62	4.63	*Psychrobacter cryohalolentis* (strain K5)
24	50S ribosomal protein L6	1	1	8.47%	18981.86	9.68	*Bordetella petrii* (strain ATCC BAA-461 / DSM 12804 / CCUG 43448)
25	V-type ATP synthase subunit D	1	1	8.45%	24772.66	8.5	*Clostridium botulinum* (strain Alaska E43 / Type E3)
26	ATP synthase gamma chain	1	1	8.03%	32437.35	9.7	*Rhodospirillum rubrum* (strain ATCC 11170 / NCIB 8255)
27	Acetylglutamate/acetylaminoadipate kinase	1	1	7.63%	25358.23	5.05	*Deinococcus radiodurans* (strain ATCC 13939 / DSM 20539 / JCM 16871 / LMG 4051 / NBRC 15346 / NCIMB 9279 / R1 / VKM B-1422)
28	4-hydroxy-tetrahydrodipicolinate synthase	1	1	7.53%	31115.87	5.55	*Dechloromonas aromatica* (strain RCB)
29	Leucyl/phenylalanyl-tRNA--protein transferase	1	1	7.52%	25671.37	7.05	*Pseudomonas aeruginosa* (strain PA7)
30	Ornithine carbamoyltransferase (Fragment)	1	1	7.33%	25768.36	5.21	*Neisseria subflava*
31	Electron transfer flavoprotein subunit beta	1	1	7.14%	28159.99	4.73	*Mycobacterium leprae* (strain TN)
32	Thiazole synthase	1	1	7.06%	27286.56	4.84	*Staphylococcus carnosus* (strain TM300)
33	Putative ankyrin repeat protein	1	1	7.01%	23510	5.73	*Rickettsia felis* (strain ATCC VR-1525 / URRWXCal2)

	RF_1081						
34	Porphobilinogen deaminase	1	1	6.80%	31073.49	4.98	*Corynebacterium diphtheriae* (strain ATCC 700971 / NCTC 13129 / Biotype gravis)
35	4-hydroxybenzoate octaprenyltransferase	1	1	6.76%	33186.2	9.1	*Pseudomonas mendocina* (strain ymp)
36	Stage 0 sporulation protein A homolog	1	1	6.59%	30683.48	6.02	*Clostridium beijerinckii* (strain ATCC 51743 / NCIMB 8052)
37	30S ribosomal protein S3	1	1	6.32%	28696.05	10.19	*Chlorobium chlorochromatii* (strain CaD3)
38	L-lactate dehydrogenase	1	1	6.29%	34974.97	5.54	*Selenomonas ruminantium*
39	4-hydroxy-3-methylbut-2-en-1-yl diphosphate synthase	1	1	6.19%	40792.81	5.13	*Arthrobacter aurescens* (strain TC1)
40	Shikimate dehydrogenase	1	1	6.19%	31946.88	8.67	*Desulfotomaculum reducens* (strain MI-1)
41	tRNA pseudouridine synthase A	1	1	6.14%	30244.48	9.09	*Desulfovibrio vulgaris* (strain Miyazaki F / DSM 19637)
42	Phosphate import ATP-binding protein PstB	1	1	5.97%	30256.63	5.49	*Gluconobacter oxydans* (strain 621H)
43	60 kDa chaperonin	4	2	5.85%	57158.67	5.02	*Delftia acidovorans* (strain DSM 14801 / SPH-1)
44	GTPase Obg	1	1	5.83%	44643.82	4.72	*Magnetospirillum magneticum* (strain AMB-1 / ATCC 700264)
45	tRNA pseudouridine synthase B	1	1	5.80%	37571.87	8.6	*Bradyrhizobium diazoefficiens* (strain JCM 10833 / IAM 13628 / NBRC 14792 / USDA 110)
46	Succinyl-CoA ligase [ADP-forming] subunit beta	1	1	5.76%	42407.96	5.34	*Sphingomonas wittichii* (strain RW1 / DSM 6014 / JCM 10273)
47	Chorismate synthase	1	1	5.71%	38673.51	8.71	*Thermoanaerobacter tengcongensis* (strain DSM 15242 / JCM 11007 / NBRC 100824 / MB4)
48	Ketol-acid reductoisomerase	1	1	5.64%	36283.49	4.77	*Corynebacterium diphtheriae* (strain ATCC 700971 / NCTC 13129 / Biotype gravis)
49	Aspartate carbamoyltransferase	1	1	5.62%	34470.94	6.86	*Xylella fastidiosa* (strain Temecula1 / ATCC 700964)

50	Biotin synthase	1	1	5.37%	36154.3	4.88	*Acidothermus cellulolyticus* (strain ATCC 43068 / 11B)
51	4-deoxy-L-threo-5-hexosulose-uronate ketol-isomerase	1	1	5.36%	31323.64	5.61	*Phenylobacterium zucineum* (strain HLK1)
52	Cytoskeleton protein RodZ	1	1	5.33%	36694.91	5.66	*Cronobacter sakazakii* (strain ATCC BAA-894)
53	Holliday junction ATP-dependent DNA helicase RuvB	1	1	5.31%	37725.77	5.43	*Carboxydothermus hydrogenoformans* (strain Z-2901 / DSM 6008)
54	Bifunctional protein FolD	1	1	5.28%	32356.81	6.67	*Desulfococcus oleovorans* (strain DSM 6200 / Hxd3)
55	tRNA N6-adenosine threonylcarbamoyltransferase	1	1	5.28%	36475.11	5.23	*Pseudomonas fluorescens* (strain Pf-5 / ATCC BAA-477)
56	60 kDa chaperonin 1	4	2	5.25%	57628.24	5.26	*Rhodoferax ferrireducens* (strain DSM 15236 / ATCC BAA-621 / T118)
57	sn-glycerol-3-phosphate import ATP-binding protein UgpC	1	1	5.15%	39783	6.91	*Cupriavidus pinatubonensis* (strain JMP 134 / LMG 1197)
58	Putative pyrophosphorylase ModD	1	1	4.98%	31436.24	5.75	*Haemophilus influenzae* (strain ATCC 51907 / DSM 11121 / KW20 / Rd)
59	Argininosuccinate lyase	1	1	4.90%	51298.6	5.73	*Burkholderia cenocepacia* (strain MC0-3)
60	tRNA dimethylallyltransferase 1	1	1	4.90%	35961.61	8.82	*Azobacteroides pseudotrichonymphae genomovar.* CFP2
61	Putative uncharacterized protein YkfC	1	1	4.79%	43165.29	10.32	*Escherichia coli* (strain K12)
62	Probable transcriptional regulatory protein Aave_3203	1	1	4.60%	25840.96	4.87	*Acidovorax citrulli* (strain AAC00-1)
63	HTH-type transcriptional regulator EbgR	1	1	4.59%	36210.45	5.69	*Escherichia coli* (strain K12)
64	Cobyric acid synthase	1	1	4.57%	55343.74	6.38	*Geobacillus kaustophilus* (strain HTA426)
65	Probable transcriptional regulatory protein HAPS_0943	1	1	4.47%	26252.18	4.66	*Haemophilus parasuis serovar 5* (strain SH0165)

66	Pyridoxal biosynthesis lyase PdxS	1	1	4.41%	31915.67	5.26	*Staphylococcus haemolyticus* (strain JCSC1435)
67	N-succinylglutamate 5-semialdehyde dehydrogenase	1	1	4.30%	51332.54	5.65	*Pseudomonas syringae* pv. *syringae* (strain B728a)
68	Deoxyhypusine synthase-like protein	1	1	4.26%	39570.03	5.49	*Coxiella burnetii* (strain Dugway 5J108-111)
69	ATP-dependent RNA helicase RhlE	1	1	4.19%	49989.37	10.06	*Escherichia coli* (strain K12)
70	Sensor histidine kinase LiaS	1	1	4.17%	40704.37	7.04	*Bacillus subtilis* (strain 168)
71	Heat-inducible transcription repressor HrcA	1	1	4.13%	36934.53	5.97	*Acidothermus cellulolyticus* (strain ATCC 43068 / 11B)
72	Serine hydroxymethyltransferase	1	1	4.08%	45488.57	5.8	*Erwinia tasmaniensis* (strain DSM 17950 / Et1/99)
73	Exodeoxyribonuclease 7 large subunit	1	1	4.04%	50536.39	9.36	*Streptococcus pneumoniae serotype* 2 (strain D39 / NCTC 7466)
74	Heptaprenyl diphosphate synthase component 2	1	1	4.02%	39515.87	6.28	*Bacillus subtilis* (strain 168)
75	Sulfate adenylyltransferase subunit 2	1	1	3.97%	34841.68	6.11	*Shewanella amazonensis* (strain ATCC BAA-1098 / SB2B)
76	Enolase	1	1	3.92%	47015.83	4.68	*Streptococcus intermedius*
77	ATPase	1	1	3.92%	49903.88	5.9	*Gemmatimonas aurantiaca* (strain T-27 / DSM 14586 / JCM 11422 / NBRC 100505)
78	Phosphoserine aminotransferase	1	1	3.88%	39967.46	5.34	*Idiomarina loihiensis* (strain ATCC BAA-735 / DSM 15497 / L2-TR)
79	Phenylalanine--tRNA ligase alpha subunit	1	1	3.61%	40097.72	5.45	*Bradyrhizobium diazoefficiens* (strain JCM 10833 / IAM 13628 / NBRC 14792 / USDA 110)
80	Uncharacterized protein YxeQ	1	1	3.60%	47690.26	5.81	*Bacillus subtilis* (strain 168)
81	Formate--tetrahydrofolate ligase	1	1	3.58%	63626.12	5.56	*Desulfatibacillum alkenivorans* (strain AK-01)

82	UDP-N-acetylglucosamine--N-acetylmuramyl-(pentapeptide) pyrophosphoryl-undecaprenol N-acetylglucosamine transferase	1	1	3.56%	40232.29	9.41	*Chloroherpeton thalassium* (strain ATCC 35110 / GB-78)
83	Mesentericin Y105 secretion protein MesE	1	1	3.50%	51155.29	8.82	*Leuconostoc mesenteroides*
84	Probable cytosol aminopeptidase	1	1	3.40%	52730.49	8.97	*Saccharophagus degradans* (strain 2-40 / ATCC 43961 / DSM 17024)
85	ATP-dependent protease ATPase subunit HslU	1	1	3.36%	49597.62	5.49	*Acidovorax ebreus* (strain TPSY)
86	Glutamine--tRNA ligase	1	1	3.35%	64762.21	5.58	*Gemmatimonas aurantiaca* (strain T-27 / DSM 14586 / JCM 11422 / NBRC 100505)
87	Transcription termination factor Rho	1	1	3.34%	46862.26	8.38	*Buchnera aphidicola* subsp. *Schizaphis graminum* (strain Sg)
88	GMP synthase [glutamine-hydrolyzing]	1	1	3.31%	56882.64	6.59	*Pelodictyon luteolum* (strain DSM 273)
89	Bifunctional protein GlmU	1	1	3.30%	49920.72	5.95	*Clostridium botulinum* (strain Alaska E43 / Type E3)
90	Glucose-6-phosphate isomerase	1	1	3.21%	58240.27	5.93	*Geobacter* sp. (strain M21)
91	Glutamine synthetase (Fragment)	2	1	3.20%	48391.18	5.25	*Rhodobacter capsulatus*
92	3-phosphoshikimate 1-carboxyvinyltransferase	1	1	3.17%	46389.03	5.58	*Gluconobacter oxydans* (strain 621H)
93	tRNA 5-methylaminomethyl-2-thiouridine biosynthesis bifunctional protein MnmC	1	1	3.13%	68617.98	6.74	*Acidovorax* sp. (strain JS42)
94	Aspartate--tRNA ligase	1	1	3.08%	66513.37	5.37	*Chlamydophila caviae* (strain GPIC)

95	Formate--tetrahydrofolate ligase 2	1	1	3.05%	59065.87	5.77	*Streptococcus pyogenes serotype* M2 (strain MGAS10270)
96	Glutamyl-tRNA reductase	1	1	3.02%	52035.9	5.27	*Corynebacterium jeikeium* (strain K411)
97	Dihydroxy-acid dehydratase	1	1	2.95%	65310.44	5.67	*Brucella abortus* (strain S19)
98	Aspartyl/glutamyl-tRNA(Asn/Gln) amidotransferase subunit B	1	1	2.94%	53352.16	5.7	*Oenococcus oeni* (strain ATCC BAA-331 / PSU-1)
99	1-deoxy-D-xylulose-5-phosphate synthase 1	1	1	2.93%	68586.33	6.22	*Rhodobacter sphaeroides* (strain ATCC 17023 / 2.4.1 / NCIB 8253 / DSM 158)
100	DNA ligase	1	1	2.93%	78968.67	5.6	*Rhizobium meliloti* (strain 1021)
101	UvrABC system protein C	1	1	2.89%	68736.51	8.83	*Rhodobacter sphaeroides* (strain ATCC 17023 / 2.4.1 / NCIB 8253 / DSM 158)
102	Cysteine--tRNA ligase	1	1	2.88%	55740.73	5.41	*Desulfotomaculum reducens* (strain MI-1)
103	tRNA uridine 5-carboxymethylaminomethyl modification enzyme MnmG	1	1	2.83%	69396.58	8.5	*Anaplasma marginale* (strain Florida)
104	Foldase protein PrsA	1	1	2.81%	35637.61	9.01	*Staphylococcus aureus* (strain Mu3 / ATCC 700698)
105	3-ketoacyl-CoA thiolase	1	1	2.81%	41644.93	6.39	*Pseudomonas oleovorans*
106	Threonine--tRNA ligase	1	1	2.80%	73849.07	5.8	*Actinobacillus succinogenes* (strain ATCC 55618 / 130Z)
107	Elongation factor G	1	1	2.60%	76117.61	5.22	*Methylobacterium radiotolerans* (strain ATCC 27329 / DSM 1819 / JCM 2831)
108	Protein TolB	1	1	2.59%	46551.68	8.8	*Nitrosomonas europaea* (strain ATCC 19718 / NBRC 14298)
109	Glutamine synthetase	1	1	2.47%	82824.68	5.88	*Bacteroides fragilis* (strain YCH46)
110	Chaperone protein HscA	1	1	2.44%	65713.6	5.26	*Erwinia carotovora* subsp. *atroseptica* (strain SCRI 1043 / ATCC BAA-672)
111	Polyribonucleotide nucleotidyltransferase	1	1	2.43%	75903.76	5.37	*Nitrosospira multiformis* (strain ATCC 25196 / NCIMB 11849)
112	DNA mismatch repair protein	1	1	2.40%	97458.63	5.35	*Prosthecochloris vibrioformis* (strain DSM 265)

	MutS						
113	ATP synthase subunit alpha	3	1	2.39%	54551.68	5.68	*Geobacter daltonii* (strain DSM 22248 / JCM 15807 / FRC-32)
114	Bifunctional purine biosynthesis protein PurH	1	1	2.27%	56291.72	4.92	*Ruegeria* sp. (strain TM1040)
115	UDP-N-acetylmuramoyl-L-alanyl-D-glutamate--2,6-diaminopimelate ligase	1	1	2.26%	53279.24	6.24	*Cytophaga hutchinsonii* (strain ATCC 33406 / NCIMB 9469)
116	(Dimethylallyl)adenosine tRNA methylthiotransferase MiaB	1	1	2.25%	48206.03	5.65	*Sphingopyxis alaskensis* (strain DSM 13593 / LMG 18877 / RB2256)
117	Polyphosphate kinase	1	1	2.17%	82692.6	6.56	*Pseudomonas syringae* pv. *tomato* (strain DC3000)
118	Alanine--tRNA ligase	1	1	2.10%	98634.42	5.58	*Roseiflexus* sp. (strain RS-1)
119	Uncharacterized protein	1	1	1.96%	126975.6	8.67	*Gemmatimonas aurantiaca* (strain T-27 / DSM 14586 / JCM 11422 / NBRC 100505)
120	Valine--tRNA ligase	1	1	1.70%	102021.1	5.6	*Clostridium acetobutylicum* (strain ATCC 824 / DSM 792 / JCM 1419 / LMG 5710 / VKM B-1787)
121	UPF0192 protein DR_A0293	1	1	1.68%	106829.5	9.69	*Deinococcus radiodurans* (strain ATCC 13939 / DSM 20539 / JCM 16871 / LMG 4051 / NBRC 15346 / NCIMB 9279 / R1 / VKM B-1422)
122	Acetyl-coenzyme A synthetase	1	1	1.64%	74143.39	5.07	*Rhodopirellula baltica* (strain SH1)
123	Translation initiation factor IF-2	1	1	1.63%	114865.9	6.47	*Bacteroides thetaiotaomicron* (strain ATCC 29148 / DSM 2079 / NCTC 10582 / E50 / VPI-5482)
124	Transcription-repair-coupling factor	1	1	1.58%	132665.8	6.27	*Myxococcus xanthus*
125	Hydroxylamine oxidoreductase	1	1	1.58%	64258.64	6.59	*Nitrosomonas europaea* (strain ATCC 19718 / NBRC 14298)
126	Chaperone protein ClpB	1	1	1.52%	95662.78	5.8	*Photorhabdus luminescens* subsp. *laumondii* (strain TT01)
127	Phosphoribosylformylglycinamidi	1	1	1.47%	140598.7	5.09	*Shewanella oneidensis* (strain MR-1)

	ne synthase						
128	DNA-directed RNA polymerase subunit beta	1	1	1.18%	151306.1	5.73	*Pseudomonas putida*
129	Bifunctional hemolysin/adenylate cyclase	1	1	0.70%	177055.9	4.59	*Bordetella bronchiseptica* (strain ATCC BAA-588 / NCTC 13252 / RB50)
130	Adenosine monophosphate-protein transferase and cysteine protease IbpA	1	1	0.42%	450059.3	5.5	*Histophilus somni* (strain 2336)

Table S3. The classified extracellular proteins in anoxic sludge analyzed by shotgun method.

No.	Protein description	Peptide counts	Unique peptide counts	Cover percent	Molecular mass	PI	Microorganism species
1	50S ribosomal protein L36	1	1	31.58%	4427.43	11.06	*Enterococcus faecalis* (strain ATCC 700802 / V583)
2	Glutaredoxin-1	1	1	22.45%	11160.94	5.65	*Rickettsia bellii* (strain RML369-C)
3	Probable Sec-independent protein translocase protein TatE	1	1	22.06%	7257.43	8.01	*Klebsiella pneumoniae* subsp. *pneumoniae* (strain ATCC 700721 / MGH 78578)
4	50S ribosomal protein L29	1	1	20.27%	8402.62	5.85	*Streptomyces avermitilis* (strain ATCC 31267 / DSM 46492 / JCM 5070 / NCIMB 12804 / NRRL 8165 / MA-4680)
5	30S ribosomal protein S18	1	1	19.54%	10143.85	10.66	*Chlamydophila abortus* (strain DSM 27085 / S26/3)
6	DNA-directed RNA polymerase subunit omega	1	1	17.58%	10176.53	4.66	*Actinobacillus pleuropneumoniae serotype* 5b (strain L20)
7	50S ribosomal protein L19	1	1	14.78%	13152.22	10.93	*Streptococcus mutans serotype* c (strain ATCC 700610 / UA159)
8	30S ribosomal protein S8	1	1	13.85%	14263.32	9.51	*Ruegeria pomeroyi* (strain ATCC 700808 / DSM 15171 / DSS-3)
9	50S ribosomal protein L18	1	1	13.27%	12644.68	10.31	*Bacteroides vulgatus* (strain ATCC 8482 / DSM 1447 / NCTC 11154)
10	30S ribosomal protein S11	1	1	13.18%	13710.64	11.19	*Jannaschia* sp. (strain CCS1)
11	Putative regulator of ribonuclease activity	1	1	12.50%	16576.67	6.04	*Rhodospirillum centenum* (strain ATCC 51521 / SW)
12	50S ribosomal protein L15	1	1	11.88%	16657.2	10.11	*Beijerinckia indica* subsp. *indica* (strain ATCC 9039 / DSM 1715 / NCIB 8712)
13	Carbon monoxide dehydrogenase small chain	1	1	11.04%	17752.42	8.08	*Hydrogenophaga pseudoflava*
14	60 kDa chaperonin 1	4	4	10.99%	57197.43	5.09	*Burkholderia xenovorans* (strain LB400)
15	60 kDa chaperonin	4	4	10.18%	57849.48	5.19	*Dechloromonas aromatica* (strain RCB)

16	Malate dehydrogenase	2	2	10.06%	35000.12	6.24	*Dechloromonas aromatica* (strain RCB)
17	30S ribosomal protein S5	1	1	9.71%	18826.91	10.41	*Rickettsia prowazekii* (strain Madrid E)
18	General secretion pathway protein B	1	1	9.55%	23486.16	5.21	*Dickeya dadantii* (strain 3937)
19	4-diphosphocytidyl-2-C-methyl-D-erythritol kinase	1	1	9.29%	31304.33	8.95	*Clostridium acetobutylicum* (strain ATCC 824 / DSM 792 / JCM 1419 / LMG 5710 / VKM B-1787)
20	Recombination protein RecR	1	1	8.96%	20952.15	6.2	*Hyphomonas neptunium* (strain ATCC 15444)
21	Serine-protein kinase RsbW	1	1	8.81%	17931.31	4.72	*Staphylococcus aureus*
22	UPF0251 protein CT1277	1	1	8.63%	21345.16	9.01	*Chlorobium tepidum* (strain ATCC 49652 / DSM 12025 / TLS)
23	3,4-dihydroxy-2-butanone 4-phosphate synthase	1	1	8.37%	23279.53	5.02	*Desulfatibacillum alkenivorans* (strain AK-01) GN=ribB PE=3 SV=1
24	Bifunctional protein PyrR	1	1	8.33%	21001.84	5.31	*Corynebacterium efficiens* (strain DSM 44549 / YS-314 / AJ 12310 / JCM 11189 / NBRC 100395)
25	7-cyano-7-deazaguanine synthase	1	1	8.04%	23698	5.27	*Pseudomonas mendocina* (strain ymp)
26	Glyceraldehyde-3-phosphate dehydrogenase (Fragment)	2	1	7.82%	31393.54	5.81	*Escherichia hermannii*
27	50S ribosomal protein L2	1	1	7.22%	30145.67	11.28	*Thermobifida fusca* (strain YX)
28	Indole-3-glycerol phosphate synthase	1	1	7.06%	29210.88	6.72	*Roseiflexus castenholzii* (strain DSM 13941 / HLO8)
29	Glyceraldehyde-3-phosphate dehydrogenase A	7	1	6.95%	35540.31	6.61	*Escherichia fergusonii* (strain ATCC 35469 / DSM 13698 / CDC 0568-73)
30	Putative aminoacrylate hydrolase RutD	1	1	6.91%	30097.26	5.32	*Pantoea ananatis* (strain LMG 20103)
31	Ribosomal RNA small subunit methyltransferase A	1	1	6.60%	30636.51	8.95	*Xanthobacter autotrophicus* (strain ATCC BAA-1158 / Py2)

32	ATP synthase subunit b	1	1	6.45%	20552.36	4.78	*Corynebacterium urealyticum* (strain ATCC 43042 / DSM 7109)
33	Ribosomal RNA small subunit methyltransferase H	1	1	6.33%	35656.74	8.07	*Cupriavidus pinatubonensis* (strain JMP 134 / LMG 1197)
34	Release factor glutamine methyltransferase	1	1	6.12%	31843.65	4.74	*Bacteroides thetaiotaomicron* (strain ATCC 29148 / DSM 2079 / NCTC 10582 / E50 / VPI-5482)
35	UDP-N-acetylglucosamine--N-acetylmuramyl-(pentapeptide) pyrophosphoryl-undecaprenol N-acetylglucosamine transferase	1	1	6.09%	37893.05	9.63	*Aeromonas hydrophila* subsp. *hydrophila* (strain ATCC 7966 / NCIB 9240)
36	DNA repair protein RecO	1	1	5.93%	27747.79	7.18	*Rhizobium loti* (strain MAFF303099)
37	Isopentenyl-diphosphate delta-isomerase	1	1	5.85%	37841.3	6.46	*Legionella pneumophila* (strain Paris)
38	Ribonuclease Z	1	1	5.56%	34606.47	5.83	*Oceanobacillus iheyensis* (strain DSM 14371 / JCM 11309 / KCTC 3954 / HTE831)
39	N-acetyl-gamma-glutamyl-phosphate reductase	1	1	5.56%	34698.51	5.65	*Frankia* sp. (strain CcI3)
40	Probable chromosome-partitioning protein ParB	1	1	5.52%	33166.45	8.4	*Helicobacter pylori* (strain ATCC 700392 / 26695)
41	UPF0042 nucleotide-binding protein Ccel_2290	1	1	5.50%	32793.18	6.98	*Clostridium cellulolyticum* (strain ATCC 35319 / DSM 5812 / JCM 6584 / H10)
42	Nicotinate-nucleotide--dimethylbenzimidazole phosphoribosyltransferase	1	1	5.41%	36153.63	6.14	*Chloroflexus aurantiacus* (strain ATCC 29366 / DSM 635 / J-10-fl)
43	Tyrosine recombinase XerC	1	1	5.25%	36240.32	10.29	*Gemmatimonas aurantiaca* (strain T-27 / DSM 14586 / JCM 11422 / NBRC 100505)

44	D-alanine--D-alanine ligase	1	1	5.19%	33615.29	5.7	*Rhizobium meliloti* (strain 1021)
45	Peptide chain release factor 1	1	1	5.07%	39961.3	5.28	*Wolinella succinogenes* (strain ATCC 29543 / DSM 1740 / LMG 7466 / NCTC 11488 / FDC 602W)
46	Urease accessory protein UreD	1	1	5.05%	30508.25	6.73	*Pseudomonas putida* (strain W619)
47	UDP-glucose 6-dehydrogenase	1	1	5.03%	47121.68	5.7	*Rhizobium meliloti* (strain 1021)
48	Molybdopterin molybdenumtransferase	1	1	5.01%	45612.52	7.11	*Staphylococcus epidermidis* (strain ATCC 35984 / RP62A)
49	Ribosomal protein S6 modification protein	1	1	5.00%	32447.39	6.72	*Enterobacter* sp. (strain 638)
50	tRNA N6-adenosine threonylcarbamoyltransferase	1	1	4.99%	36395.06	5.54	*Pseudomonas fluorescens* (strain SBW25)
51	Phosphatidylserine decarboxylase proenzyme	1	1	4.86%	31799.99	9.09	*Bordetella petrii* (strain ATCC BAA-461 / DSM 12804 / CCUG 43448)
52	Diaminopimelate epimerase	1	1	4.84%	30997.08	5.44	*Burkholderia vietnamiensis* (strain G4 / LMG 22486)
53	Succinyl-diaminopimelate desuccinylase	1	1	4.81%	42767.45	5.02	*Mesorhizobium* sp. (strain BNC1)
54	HTH-type transcriptional regulator rgg	1	1	4.71%	34439.46	5.86	*Streptococcus gordonii* (strain Challis / ATCC 35105 / CH1 / DL1 / V288)
55	UDP-N-acetylmuramate--L-alanine ligase	1	1	4.57%	52136.09	5.29	*Marinobacter aquaeolei* (strain ATCC 700491 / DSM 11845 / VT8)
56	Orotidine 5'-phosphate decarboxylase	1	1	4.35%	30572.03	5.03	*Porphyromonas gingivalis* (strain ATCC BAA-308 / W83)
57	N-methyl-L-tryptophan oxidase	1	1	4.30%	40772.07	5.86	*Salmonella typhi*
58	Ornithine carbamoyltransferase	1	1	4.22%	34100.83	5.46	*Roseobacter denitrificans* (strain ATCC 33942 / OCh 114)
59	Aminomethyltransferase	1	1	4.16%	39869.6	5.4	*Bacteroides fragilis* (strain ATCC 25285 / NCTC 9343)
60	Phosphoglycerate kinase	1	1	4.08%	43040.56	4.7	*Renibacterium salmoninarum* (strain ATCC 33209 / DSM 20767 /

							JCM 11484 / NBRC 15589 / NCIMB 2235)
61	UDP-N-acetylglucosamine 1-carboxyvinyltransferase	1	1	4.06%	45472.07	8.8	*Rickettsia prowazekii* (strain Madrid E)
62	Chaperone protein DnaK 2	1	1	4.06%	69250.67	4.67	*Photobacterium profundum*
63	Serine--tRNA ligase	1	1	3.99%	46801.1	5.13	*Arthrobacter aurescens* (strain TC1)
64	Arginine--tRNA ligase	1	1	3.96%	62629.34	4.93	*Staphylococcus saprophyticus* subsp. *saprophyticus* (strain ATCC 15305 / DSM 20229)
65	ESX-1 secretion-associated protein EspB	1	1	3.96%	46972.97	4.52	*Mycobacterium marinum* (strain ATCC BAA-535 / M)
66	Argininosuccinate lyase	1	1	3.81%	50607.42	5.64	*Bacteroides vulgatus* (strain ATCC 8482 / DSM 1447 / NCTC 11154)
67	Holliday junction ATP-dependent DNA helicase RuvB	1	1	3.71%	38302.84	5.44	*Rhodospirillum rubrum* (strain ATCC 11170 / NCIB 8255)
68	GTPase HflX	1	1	3.66%	48734.06	5.54	*Akkermansia muciniphila* (strain ATCC BAA-835)
69	Trigger factor	1	1	3.62%	50710	5.4	*Helicobacter hepaticus* (strain ATCC 51449 / 3B1)
70	3-phosphoshikimate 1-carboxyvinyltransferase	1	1	3.60%	46446.09	5.21	*Halorhodospira halophila* (strain DSM 244 / SL1)
71	Histidine--tRNA ligase	1	1	3.53%	47556.38	6.35	*Heliobacterium modesticaldum* (strain ATCC 51547 / Ice1)
72	Aspartate--tRNA ligase	1	1	3.53%	64858.28	5.51	*Acidiphilium cryptum* (strain JF-5)
73	Elongation factor Tu	1	1	3.27%	43912.26	5.3	*Syntrophus aciditrophicus* (strain SB)
74	Adenylosuccinate synthetase	1	1	3.25%	46317.72	5.21	*Mycobacterium gilvum* (strain PYR-GCK)
75	GMP synthase [glutamine-hydrolyzing]	1	1	3.23%	58532.85	6.15	*Ehrlichia canis* (strain Jake)
76	UPF0226 protein PA1993	1	1	3.23%	41455.16	10.08	*Pseudomonas aeruginosa* (strain ATCC 15692 / PAO1 / 1C / PRS 101 / LMG 12228)

77	sp|P13499|GLNA_RHOCA Glutamine synthetase (Fragment)	1	1	3.20%	48391.18	5.25	*Rhodobacter capsulatus*
78	Phosphoribosylformylglycinamidine synthase 2	1	1	3.12%	78207.52	5.27	*Bradyrhizobium* sp. (strain ORS278)
79	Acetylornithine aminotransferase	1	1	2.98%	43515.86	5.69	*Vibrio vulnificus* (strain CMCP6)
80	D-inositol 3-phosphate glycosyltransferase	1	1	2.80%	45291.8	7.25	*Mycobacterium leprae* (strain Br4923)
81	UvrABC system protein C	1	1	2.77%	77280.7	6.68	*Corynebacterium diphtheriae* (strain ATCC 700971 / NCTC 13129 / Biotype gravis)
82	Anaerobic nitric oxide reductase transcription regulator NorR	1	1	2.73%	56767.28	5.96	*Vibrio fischeri* (strain ATCC 700601 / ES114)
83	Elongation factor G 2	1	1	2.62%	72921.44	5.86	*Streptomyces coelicolor* (strain ATCC BAA-471 / A3(2) / M145)
84	1-deoxy-D-xylulose-5-phosphate synthase	1	1	2.62%	70287.22	5.28	*Clavibacter michiganensis* subsp. *sepedonicus* (strain ATCC 33113 / DSM 20744 / JCM 9667 / LMG 2889 / C-1)
85	C4-dicarboxylate transport protein	1	1	2.49%	47321.46	8.55	*Polynucleobacter necessarius* subsp. *asymbioticus* (strain DSM 18221 / CIP 109841 / QLW-P1DMWA-1)
86	Polyribonucleotide nucleotidyltransferase	1	1	2.38%	76634.58	5.02	*Methylobacillus flagellatus* (strain KT / ATCC 51484 / DSM 6875)
87	ATP synthase subunit beta	1	1	2.37%	50771.26	5.11	*Protochlamydia amoebophila* (strain UWE25)
88	Lon protease	1	1	2.36%	92920.01	5.14	*Gemmatimonas aurantiaca* (strain T-27 / DSM 14586 / JCM 11422 / NBRC 100505)
89	6-phosphogluconate dehydrogenase, decarboxylating	1	1	2.35%	52645.74	9.02	*Buchnera aphidicola* subsp. *Baizongia pistaciae* (strain Bp)
90	GTP-binding protein	1	1	2.30%	69246.54	8.89	*Buchnera aphidicola* subsp. *Schizaphis graminum* (strain Sg)

	TypA/BipA homolog						
91	Aspartyl/glutamyl-tRNA(Asn/Gln) amidotransferase subunit B	1	1	2.30%	52880.99	4.91	*Alkalilimnicola ehrlichii* (strain ATCC BAA-1101 / DSM 17681 / MLHE-1)
92	5-methyltetrahydropteroyltriglutamate--homocysteine methyltransferase	1	1	2.26%	84488.15	5.56	*Citrobacter koseri* (strain ATCC BAA-895 / CDC 4225-83 / SGSC4696)
93	Phosphomethylpyrimidine synthase	1	1	2.20%	75877.81	5.76	*Yersinia pestis*
94	ATP-dependent zinc metalloprotease FtsH	1	1	2.18%	69483.3	5.42	*Veillonella parvula* (strain ATCC 10790 / DSM 2008 / JCM 12972 / Te3)
95	Ferripyoverdine receptor	1	1	2.09%	91167.15	5.38	*Pseudomonas aeruginosa* (strain ATCC 15692 / PAO1 / 1C / PRS 101 / LMG 12228)
96	Phosphoenolpyruvate carboxylase	1	1	2.00%	101058.1	5.47	*Corynebacterium diphtheriae* (strain ATCC 700971 / NCTC 13129 / Biotype gravis)
97	Signal recognition particle receptor FtsY	1	1	1.88%	50508.22	4.48	*Agrobacterium tumefaciens* (strain C58 / ATCC 33970)
98	Error-prone DNA polymerase	1	1	1.86%	114629.9	7.18	*Pseudomonas aeruginosa* (strain PA7)
99	Chromosome partition protein Smc	1	1	1.63%	130653.1	5.5	*Methylococcus capsulatus* (strain ATCC 33009 / NCIMB 11132 / Bath)
100	Polyribonucleotide nucleotidyltransferase 3	1	1	1.57%	77005.04	5.27	*Alkaliphilus metalliredigens* (strain QYMF)
101	Leucine--tRNA ligase	1	1	1.49%	91634.65	5.08	*Bacillus licheniformis* (strain DSM 13 / ATCC 14580)
102	Phthiocerol/phenolphthiocerol synthesis polyketide synthase type I PpsE	1	1	1.34%	158746.5	5.4	*Mycobacterium bovis* (strain ATCC BAA-935 / AF2122/97)
103	Isoleucine--tRNA ligase	1	1	1.32%	103868	6.67	*Methylacidiphilum infernorum* (isolate V4)

104	2-oxoglutarate dehydrogenase E1 component	1	1	1.18%	106723	7.41	*Coxiella burnetii* (strain RSA 493 / Nine Mile phase I)
105	DNA-directed RNA polymerase subunit beta'	1	1	1.13%	168949	5.68	*Chloroflexus aggregans* (strain MD-66 / DSM 9485)
106	Phenolphthiocerol synthesis polyketide synthase type I Pks15/1	1	1	0.90%	217744.6	4.72	*Mycobacterium marinum* (strain ATCC BAA-535 / M)
107	Filamentous hemagglutinin	1	1	0.47%	367520.5	8.85	*Bordetella pertussis* (strain Tohama I / ATCC BAA-589 / NCTC 13251)
108	Mycosubtilin synthase subunit C	1	1	0.31%	297947.1	5.15	*Bacillus subtilis*

Table S4. The classified extracellular proteins in aerobic sludge analyzed by shotgun method.

No.	Protein description	Peptide counts	Unique peptide counts	Cover percent	Molecular mass	PI	Microorganism species
1	Translation initiation factor IF-1	1	1	15.28%	8311.7	9.45	*Dinoroseobacter shibae* (strain DFL 12)
2	50S ribosomal protein L23	2	1	15.00%	11199.06	9.94	*Escherichia coli* O157:H7
3	ATP-dependent Clp protease adapter protein ClpS	1	1	14.95%	12653.52	5.65	*Syntrophus aciditrophicus* (strain SB)
4	30S ribosomal protein S20	1	1	14.29%	9697.44	10.67	*Bacteroides vulgatus* (strain ATCC 8482 / DSM 1447 / NCTC 11154)
5	30S ribosomal protein S8	1	1	12.88%	14522.62	9.52	*Oligotropha carboxidovorans* (strain ATCC 49405 / DSM 1227 / OM5)
6	30S ribosomal protein S14	1	1	12.87%	12083.29	11.77	*Nitrosococcus oceani* (strain ATCC 19707 / NCIMB 11848)
7	50S ribosomal protein L4	1	1	11.44%	22086.47	9.72	*Cronobacter sakazakii* (strain ATCC BAA-894)
8	30S ribosomal protein S7	1	1	10.32%	17891.75	10.23	*Halorhodospira halophila* (strain DSM 244 / SL1)
9	Ribonuclease H	1	1	10.14%	16409.63	9	*Acidovorax* sp. (strain JS42)
10	50S ribosomal protein L9	1	1	10.06%	19330.22	5.44	*Porphyromonas gingivalis* (strain ATCC 33277 / DSM 20709 / JCM 12257)
11	50S ribosomal protein L15	1	1	9.88%	16860.36	10.69	*Rhodospirillum rubrum* (strain ATCC 11170 / NCIB 8255)
12	50S ribosomal protein L3	1	1	8.88%	22754.71	9.99	*Teredinibacter turnerae* (strain ATCC 39867 / T7901)
13	50S ribosomal protein L11	1	1	8.51%	14788.2	9.56	*Lactobacillus delbrueckii* subsp. *bulgaricus* (strain ATCC 11842 / DSM 20081)
14	Uracil phosphoribosyltransferase	1	1	8.17%	22518.16	5.29	*Desulfovibrio salexigens* (strain ATCC 14822 / DSM 2638 / NCIB 8403 / VKM B-1763)

15	Protein-disulfide oxidoreductase DsbI	1	1	8.14%	24491.82	6.28	*Lelliottia amnigena*
16	50S ribosomal protein L6	1	1	7.95%	19380.24	9.59	*Desulfococcus oleovorans* (strain DSM 6200 / Hxd3)
17	Glyceraldehyde-3-phosphate dehydrogenase (Fragment)	5	1	7.82%	31393.54	5.81	*Escherichia hermannii*
18	Protease HtpX homolog	1	1	7.74%	31611.73	9.65	*Hyphomonas neptunium* (strain ATCC 15444)
19	Xanthine phosphoribosyltransferase	1	1	7.37%	21588.96	5.58	*Finegoldia magna* (strain ATCC 29328)
20	UDP-N-acetylenolpyruvoylglucosamine reductase	1	1	7.33%	32205.38	5.5	*Lactobacillus fermentum* (strain NBRC 3956 / LMG 18251)
21	Orotidine 5'-phosphate decarboxylase	1	1	7.17%	24690.52	6.43	*Rhodopseudomonas palustris* (strain ATCC BAA-98 / CGA009)
22	ATP synthase subunit b	1	1	7.10%	19314.69	9.36	*Campylobacter curvus* (strain 525.92)
23	Glyceraldehyde-3-phosphate dehydrogenase A	6	1	6.95%	35540.31	6.61	*Escherichia fergusonii* (strain ATCC 35469 / DSM 13698 / CDC 0568-73)
24	tRNA pseudouridine synthase D	1	1	6.92%	37244.46	9.22	*Anaeromyxobacter dehalogenans* (strain 2CP-C)
25	Thiazole synthase	1	1	6.92%	27701.02	5.09	*Rhodopseudomonas palustris* (strain HaA2)
26	30S ribosomal protein S4	1	1	6.90%	23184.73	10.25	*Chlorobium phaeobacteroides* (strain DSM 266)
27	Cysteine synthase A	1	1	6.81%	34489.61	5.83	*Escherichia coli* O157:H7
28	Acetylglutamate kinase	1	1	6.71%	30346.35	6.46	*Sulfurimonas denitrificans* (strain ATCC 33889 / DSM 1251)
29	Putative mannosyl-3-phosphoglycerate phosphatase	1	1	6.64%	30456.32	4.93	*Escherichia coli* O139:H28 (strain E24377A / ETEC)
30	30S ribosomal protein S2	1	1	6.30%	28853.12	5.42	*Oceanobacillus iheyensis* (strain DSM 14371 / JCM 11309 / KCTC 3954 / HTE831)
31	Hydroxyethylthiazole kinase	1	1	5.93%	28364.56	5.33	*Roseiflexus castenholzii* (strain DSM 13941 / HLO8)

32	ATP phosphoribosyltransferase	1	1	5.63%	30541.92	4.84	*Mycobacterium avium* (strain 104)
33	Biotin synthase	1	1	5.52%	36793.07	7.97	*Ehrlichia chaffeensis* (strain ATCC CRL-10679 / Arkansas)
34	Tryptophan synthase beta chain	1	1	5.37%	42983.92	5.16	*Clostridium beijerinckii* (strain ATCC 51743 / NCIMB 8052)
35	60 kDa chaperonin 2	2	2	5.31%	57418.01	5.09	*Chromobacterium violaceum* (strain ATCC 12472 / DSM 30191 / JCM 1249 / NBRC 12614 / NCIMB 9131 / NCTC 9757)
36	60 kDa chaperonin	2	2	5.31%	57479.45	5.04	*Bordetella petrii* (strain ATCC BAA-461 / DSM 12804 / CCUG 43448)
37	GTPase Obg	1	1	5.18%	43306.65	4.76	*Parabacteroides distasonis* (strain ATCC 8503 / DSM 20701 / NCTC 11152)
38	Probable septum site-determining protein MinC	1	1	5.15%	25124.62	6.59	*Proteus mirabilis* (strain HI4320)
39	Holliday junction ATP-dependent DNA helicase RuvB	1	1	5.11%	36324.66	5.39	*Deinococcus radiodurans* (strain ATCC 13939 / DSM 20539 / JCM 16871 / LMG 4051 / NBRC 15346 / NCIMB 9279 / R1 / VKM B-1422)
40	Dual-specificity RNA methyltransferase RlmN	1	1	5.01%	40295.16	9.14	*Halorhodospira halophila* (strain DSM 244 / SL1)
41	Nodulation protein G	1	1	4.88%	25717.43	5.58	*Azospirillum brasilense*
42	Malate dehydrogenase	1	1	4.86%	35031.97	6.09	*Aromatoleum aromaticum* (strain EbN1)
43	Catabolic 3-dehydroquinate dehydratase	1	1	4.78%	29898.79	9.14	*Acinetobacter baylyi* (strain ATCC 33305 / BD413 / ADP1)
44	2,3,4,5-tetrahydropyridine-2,6-dicarboxylate N-succinyltransferase	1	1	4.74%	31184.13	9.2	*Buchnera aphidicola* subsp. *Acyrthosiphon pisum* (strain APS)
45	ATP synthase gamma chain	1	1	4.62%	33793.52	9.15	*Oenococcus oeni* (strain ATCC BAA-331 / PSU-1)
46	Triosephosphate isomerase	1	1	4.44%	25274.13	5.82	*Thiobacillus denitrificans* (strain ATCC 25259)
47	Protein TolB	1	1	4.42%	46251.09	6.98	*Escherichia fergusonii* (strain ATCC 35469 / DSM 13698 / CDC

							0568-73)
48	Ribose import ATP-binding protein RbsA	1	1	4.39%	55020.42	5.76	*Erwinia carotovora* subsp. *atroseptica* (strain SCRI 1043 / ATCC BAA-672)
49	60 kDa chaperonin 1	2	2	4.37%	57884.52	5.14	*Azoarcus* sp. (strain BH72)
50	Enolase	1	1	4.28%	46583.81	5.39	*Ehrlichia canis* (strain Jake)
51	Phosphoribosylglycinamide formyltransferase 2	1	1	4.20%	43022.21	5.63	*Bordetella avium* (strain 197N)
52	Ribosomal RNA large subunit methyltransferase M	1	1	4.12%	40866.43	8.87	*Azoarcus* sp. (strain BH72)
53	Probable cytosol aminopeptidase	1	1	4.08%	50802.24	5.74	*Gluconobacter oxydans* (strain 621H)
54	Putative competence-damage inducible protein	1	1	4.05%	45076.09	5.61	*Lactobacillus plantarum* (strain ATCC BAA-793 / NCIMB 8826 / WCFS1)
55	DNA-directed RNA polymerase subunit alpha	1	1	4.03%	41393.13	5	*Ehrlichia canis* (strain Jake)
56	Uncharacterized protein	1	1	4.00%	38578.13	9.78	*Gemmatimonas aurantiaca* (strain T-27 / DSM 14586 / JCM 11422 / NBRC 100505)
57	4-hydroxy-3-methylbut-2-en-1-yl diphosphate synthase	1	1	3.97%	37369.31	5.67	*Geobacter sulfurreducens* (strain ATCC 51573 / DSM 12127 / PCA)
58	ATP synthase subunit beta	1	1	3.90%	50059.19	4.87	*Tolumonas auensis* (strain DSM 9187 / TA4)
59	23S rRNA (uracil(1939)-C(5))-methyltransferase RlmD	1	1	3.86%	51057.71	6.58	*Ralstonia metallidurans* (strain CH34 / ATCC 43123 / DSM 2839)
60	Anhydro-N-acetylmuramic acid kinase	1	1	3.75%	39814.7	6.24	*Erwinia tasmaniensis* (strain DSM 17950 / Et1/99)
61	L-lactate dehydrogenase	1	1	3.66%	40719.97	9.02	*Caulobacter crescentus* (strain NA1000 / CB15N)

	[cytochrome]						
62	GTPase HflX	1	1	3.66%	48734.06	5.54	*Akkermansia muciniphila* (strain ATCC BAA-835)
63	Histidine--tRNA ligase	1	1	3.57%	47159.09	5.92	*Anaplasma phagocytophilum* (strain HZ)
64	Argininosuccinate lyase	1	1	3.44%	51211.43	5.88	*Aromatoleum aromaticum* (strain EbN1)
65	Ribulose bisphosphate carboxylase large chain 2	1	1	3.40%	52004.98	5.56	*Hydrogenovibrio marinus*
66	4-diphosphocytidyl-2-C-methyl-D-erythritol kinase	1	1	3.38%	30698.47	7.68	*Rhodopseudomonas palustris* (strain HaA2)
67	ATP-dependent Clp protease ATP-binding subunit ClpX	1	1	3.32%	46768	6.31	*Coxiella burnetii* (strain CbuK_Q154)
68	Tyrosine--tRNA ligase	1	1	3.24%	45339.5	6.35	*Legionella pneumophila* subsp. *pneumophila* (strain Philadelphia 1 / ATCC 33152 / DSM 7513)
69	Glutamate decarboxylase	1	1	3.22%	53923.68	5.06	*Lactococcus lactis* subsp. *cremoris* (strain MG1363)
70	Glutamine synthetase (Fragment)	2	1	3.20%	48391.18	5.25	*Rhodobacter capsulatus*
71	UDP-N-acetylglucosamine--N-acetylmuramyl-(pentapeptide) pyrophosphoryl-undecaprenol N-acetylglucosamine transferase	1	1	3.11%	37781.8	9.35	*Photobacterium profundum*
72	o-succinylbenzoate synthase	1	1	3.09%	36028.32	4.58	*Aliivibrio salmonicida* (strain LFI1238)
73	Probable methylmalonyl-CoA mutase small subunit	1	1	3.09%	64744.57	5.3	*Mycobacterium bovis* (strain ATCC BAA-935 / AF2122/97)
74	ATP synthase subunit beta 1	1	1	3.04%	50149.28	4.91	*Nitrosomonas eutropha* (strain C91)
75	Glutamate-1-semialdehyde 2,1-aminomutase	1	1	2.99%	46473.44	6.47	*Coxiella burnetii* (strain CbuK_Q154)
76	Glycogen synthase	1	1	2.93%	51182.88	5.72	*Rhodobacter sphaeroides* (strain ATCC 17025 / ATH 2.4.3)

77	Catalase-peroxidase	1	1	2.75%	79388.17	5.66	*Burkholderia mallei* (strain NCTC 10247)
78	Glycerol kinase	1	1	2.62%	55435.72	4.95	*Listeria innocua serovar* 6a (strain CLIP 11262)
79	Nitrogenase iron-molybdenum cofactor biosynthesis protein NifE	1	1	2.56%	59509.16	5.75	*Bradyrhizobium diazoefficiens* (strain JCM 10833 / IAM 13628 / NBRC 14792 / USDA 110)
80	Tyrosine-protein kinase etk	1	1	2.48%	81097.57	6.48	*Escherichia coli* O127:H6 (strain E2348/69 / EPEC)
81	Aspartate--tRNA ligase	1	1	2.46%	68804.79	5.79	*Acidobacterium capsulatum* (strain ATCC 51196 / DSM 11244 / JCM 7670)
82	Chaperone protein HtpG	1	1	2.42%	70807.29	5.52	*Rickettsia akari* (strain Hartford)
83	Glycine--tRNA ligase beta subunit	1	1	2.41%	77046.22	5.65	*Rhizobium etli* (strain CFN 42 / ATCC 51251)
84	Lipoprotein LpqB	1	1	2.40%	67519.51	4.44	*Thermobifida fusca* (strain YX)
85	ATP synthase subunit alpha	1	1	2.39%	54551.68	5.68	*Geobacter daltonii* (strain DSM 22248 / JCM 15807 / FRC-32)
86	Protein translocase subunit SecA 2	1	1	2.30%	71074.2	10.03	*Ruegeria pomeroyi* (strain ATCC 700808 / DSM 15171 / DSS-3)
87	Phosphoenolpyruvate synthase	1	1	2.27%	87435.19	4.93	*Escherichia coli* (strain K12)
88	Polyribonucleotide nucleotidyltransferase	1	1	2.23%	79191.91	5.3	*Campylobacter jejuni* subsp. *doylei* (strain ATCC BAA-1458 / RM4099 / 269.97)
89	Pullulanase	1	1	2.23%	81076.96	5.83	*Bacillus subtilis* (strain 168)
90	Protein translocase subunit SecA	1	1	2.21%	103233.8	6.28	*Rickettsia africae* (strain ESF-5)
91	Alanine--tRNA ligase	1	1	2.16%	96065.14	5.44	*Pelobacter propionicus* (strain DSM 2379)
92	Isoleucine--tRNA ligase	1	1	2.10%	130111.4	5.54	*Bacteroides fragilis* (strain ATCC 25285 / NCTC 9343)
93	DNA mismatch repair protein MutL	1	1	2.07%	71654.26	6.21	*Haemophilus influenzae* (strain 86-028NP)
94	Probable protein kinase UbiB	1	1	2.02%	62599.03	8.81	*Providencia stuartii*
95	Leucine--tRNA ligase	1	1	1.97%	95873.39	5.4	*Burkholderia multivorans* (strain ATCC 17616 / 249)

96	Ribonuclease E	1	1	1.93%	106584.6	6.78	*Haemophilus influenzae* (strain ATCC 51907 / DSM 11121 / KW20 / Rd)
97	Chaperone protein DnaK	1	1	1.91%	67953.97	5.09	*Rickettsia africae* (strain ESF-5)
98	Exonuclease	1	1	1.88%	90134.58	5.2	*Gemmatimonas aurantiaca* (strain T-27 / DSM 14586 / JCM 11422 / NBRC 100505)
99	Uncharacterized RNA methyltransferase TTE1812	1	1	1.77%	50936.04	8.62	*Thermoanaerobacter tengcongensis* (strain DSM 15242 / JCM 11007 / NBRC 100824 / MB4)
100	Septation ring formation regulator EzrA	1	1	1.75%	66526.75	4.91	*Listeria innocua serovar* 6a (strain CLIP 11262)
101	D-(-)-3-hydroxybutyrate oligomer hydrolase	1	1	1.70%	72289.39	8.3	*Polaromonas naphthalenivorans* (strain CJ2)
102	Xaa-Pro dipeptidyl-peptidase	1	1	1.63%	87981.3	6.14	*Lactobacillus rhamnosus*
103	Glucose-6-phosphate isomerase	1	1	1.62%	62001.68	6.24	*Pseudomonas aeruginosa* (strain PA7)
104	DNA translocase FtsK	1	1	1.49%	88910.08	8.58	*Chlorobium tepidum* (strain ATCC 49652 / DSM 12025 / TLS)
105	Type I restriction-modification system restriction subunit	1	1	1.47%	116615	5.71	*Gemmatimonas aurantiaca* (strain T-27 / DSM 14586 / JCM 11422 / NBRC 100505)
106	Lysylphosphatidylglycerol biosynthesis bifunctional protein LysX	1	1	1.44%	129120.1	8.7	*Mycobacterium avium* (strain 104)
107	Lon protease	2	1	1.37%	87789.07	5.33	*Burkholderia phytofirmans* (strain DSM 17436 / PsJN)
108	DNA-directed RNA polymerase subunit beta	1	1	1.09%	153662.2	5.67	*Orientia tsutsugamushi* (strain Boryong)
109	DNA-directed RNA polymerase subunit beta'	1	1	1.04%	160853.4	6.45	*Gemmatimonas aurantiaca* (strain T-27 / DSM 14586 / JCM 11422 / NBRC 100505)
110	Carbamoyl-phosphate synthase large chain	1	1	1.01%	119125.1	5.03	*Gemmatimonas aurantiaca* (strain T-27 / DSM 14586 / JCM 11422 / NBRC 100505)

111	Chromosome partition protein MukB	1	1	1.01%	170188	5.22	*Escherichia coli* O139:H28 (strain E24377A / ETEC)
112	IgA-specific serine endopeptidase autotransporter	1	1	0.98%	168975.9	9.11	*Neisseria gonorrhoeae*
113	Nitrate reductase	1	1	0.92%	93897.16	8.62	*Klebsiella oxytoca*
114	Toxin A	1	1	0.77%	308055.2	5.5	*Clostridium difficile*

Table S5. The shared proteins in three sludge samples.

Sludge samples	Protein names
Anaerobic and anoxic	ATP synthase subunit b
	ATP synthase subunit beta
	Malate dehydrogenase
	Diaminopimelate epimerase
	60 kDa chaperonin
	Holliday junction ATP-dependent DNA helicase RuvB
	tRNA N6-adenosine threonylcarbamoyltransferase
	60 kDa chaperonin 1
	Argininosuccinate lyase
	UDP-N-acetylglucosamine--N-acetylmuramyl-(pentapeptide) pyrophosphoryl-undecaprenol N-acetylglucosamine transferase
	GMP synthase [glutamine-hydrolyzing]
	Glutamine synthetase (Fragment)
	3-phosphoshikimate 1-carboxyvinyltransferase
	Aspartate--tRNA ligase
	Aspartyl/glutamyl-tRNA(Asn/Gln) amidotransferase subunit B
	UvrABC system protein C
	Polyribonucleotide nucleotidyltransferase
Aerobic and anoxic	30S ribosomal protein S8
	50S ribosomal protein L15
	60 kDa chaperonin 1
	60 kDa chaperonin
	Malate dehydrogenase
	4-diphosphocytidyl-2-C-methyl-D-erythritol kinase
	Glyceraldehyde-3-phosphate dehydrogenase (Fragment)
	Glyceraldehyde-3-phosphate dehydrogenase A
	ATP synthase subunit b
	UDP-N-acetylglucosamine--N-acetylmuramyl-(pentapeptide) pyrophosphoryl-undecaprenol N-acetylglucosamine transferase
	Orotidine 5'-phosphate decarboxylase
	Argininosuccinate lyase
	Holliday junction ATP-dependent DNA helicase RuvB
	GTPase HflX
	Histidine--tRNA ligase
	Aspartate--tRNA ligase
	Glutamine synthetase (Fragment)
	Polyribonucleotide nucleotidyltransferase
	ATP synthase subunit beta
	Lon protease
	Leucine--tRNA ligase
	Isoleucine--tRNA ligase
	DNA-directed RNA polymerase subunit beta'

Anaerobic and aerobic	ATP synthase subunit b
	ATP synthase subunit beta
	Malate dehydrogenase
	50S ribosomal protein L6
	ATP synthase gamma chain
	4-hydroxy-3-methylbut-2-en-1-yl diphosphate synthase
	60 kDa chaperonin
	GTPase Obg
	Biotin synthase
	Holliday junction ATP-dependent DNA helicase RuvB
	60 kDa chaperonin 1
	Argininosuccinate lyase
	Enolase
	UDP-N-acetylglucosamine--N-acetylmuramyl-(pentapeptide)
	pyrophosphoryl-undecaprenol N-acetylglucosamine transferase
	Probable cytosol aminopeptidase
	Glucose-6-phosphate isomerase
	Glutamine synthetase (Fragment)
	Aspartate--tRNA ligase
	Polyribonucleotide nucleotidyltransferase
	ATP synthase subunit alpha
	Alanine--tRNA ligase
	Uncharacterized protein
	DNA-directed RNA polymerase subunit beta

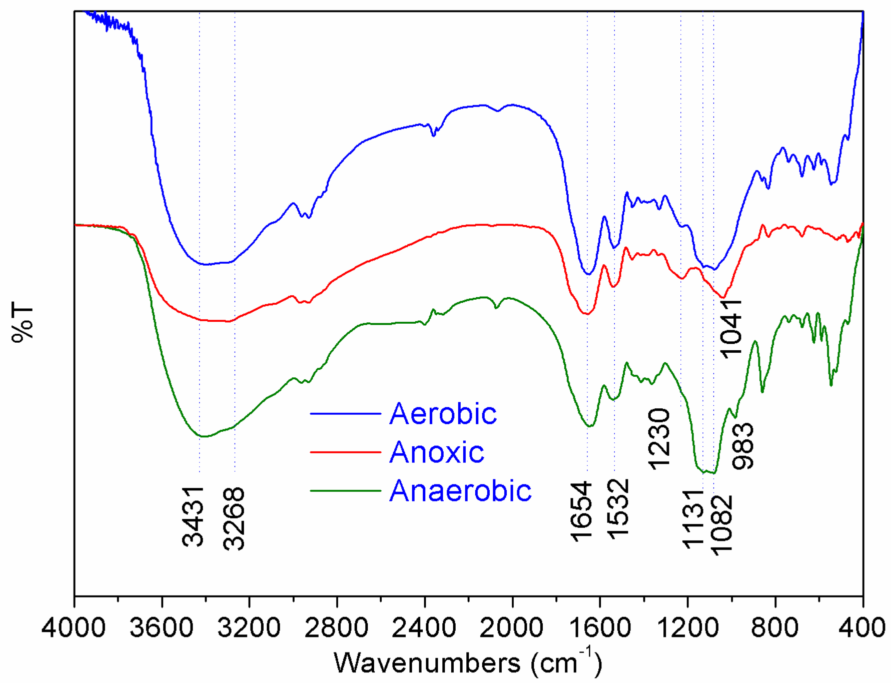

Figure S1. FT-IR spectra of extracelluar proteins from anaerobic, anoxic and aerobic sludges

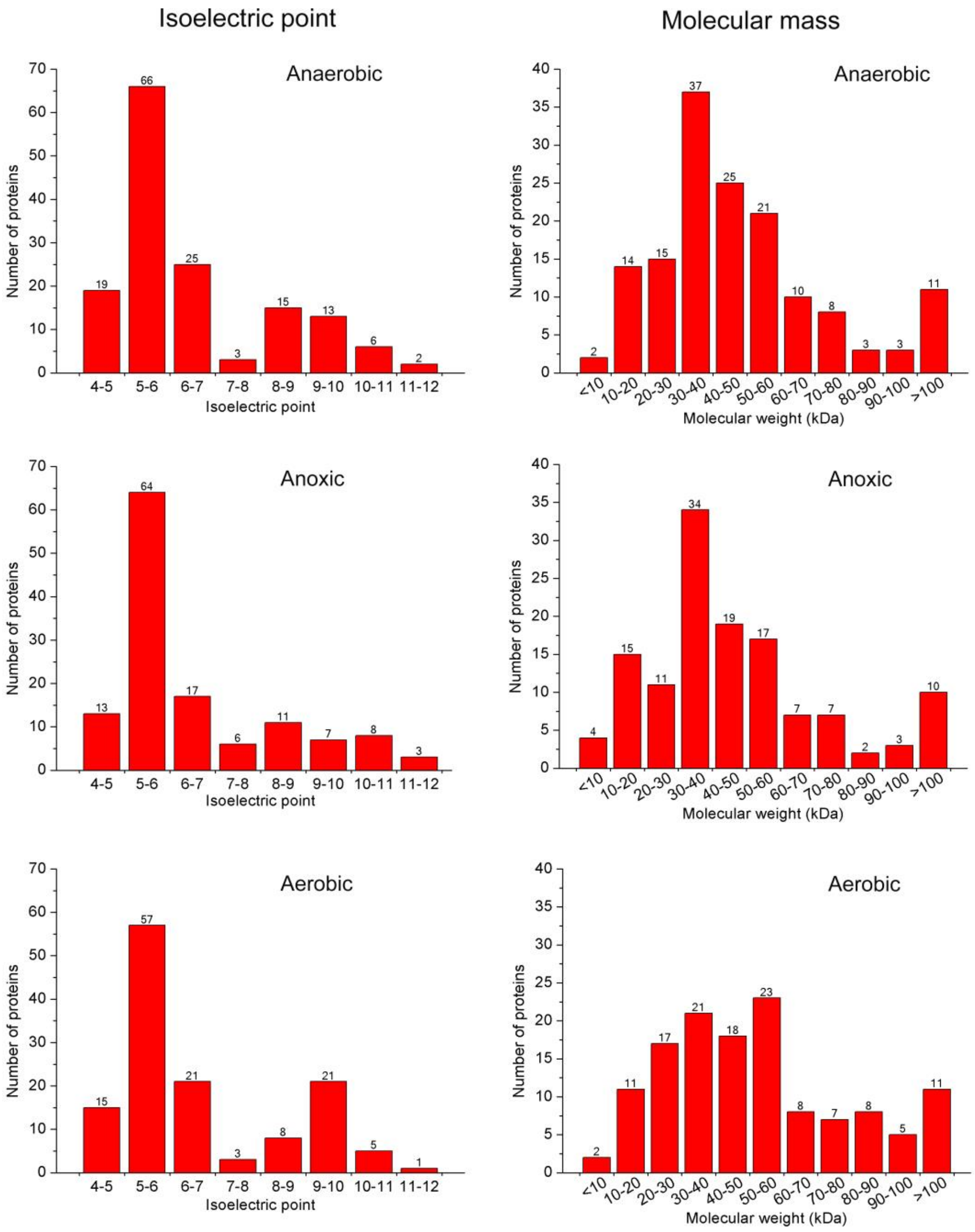

Figure S2. Distributions of pI and molecular mass of the extracelluar proteins in anaerobic, anoxic and aerobic sludges.

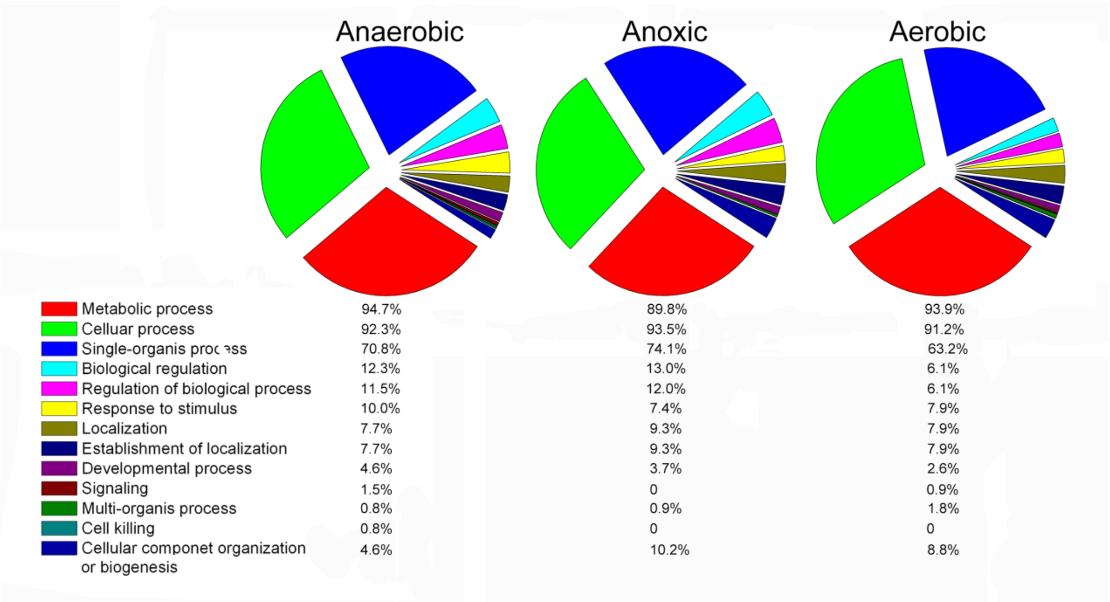

Figure S3. Proteins classification according to biological process. Numbers and percentages of the identified proteins in anaerobic, anoxic and aerobic sludge samples.
